# Height and body fatness and colorectal cancer risk: an update of the WCRF–AICR systematic review of published prospective studies

**DOI:** 10.1007/s00394-017-1557-1

**Published:** 2017-10-28

**Authors:** Leila Abar, Ana Rita Vieira, Dagfinn Aune, Jakub G. Sobiecki, Snieguole Vingeliene, Elli Polemiti, Christophe Stevens, Darren C. Greenwood, Doris S. M. Chan, Sabrina Schlesinger, Teresa Norat

**Affiliations:** 1 Department of Epidemiology and Biostatistics, School of Public Health, Imperial College London, St. Mary’s Campus, Norfolk Place, Paddington, London, W2 1PG UK; 2 Biostatistics Unit, Centre for Epidemiology and Biostatistics, University of Leeds, Leeds, UK

**Keywords:** Height, BMI, Colorectal cancer, Meta-analysis, Continuous update project

## Abstract

**Purpose:**

There is no published dose–response meta-analysis on the association between height and colorectal cancer risk (CRC) by sex and anatomical sub-site. We conducted a meta-analysis of prospective studies on the association between height and CRC risk with subgroup analysis and updated evidence on the association between body fatness and CRC risk.

**Methods:**

PubMed and several other databases were searched up to November 2016. A random effects model was used to calculate dose–response summary relative risks (RR’s).

**Results:**

47 studies were included in the meta-analyses including 50,936 cases among 7,393,510 participants. The findings support the existing evidence regarding a positive association of height, general and abdominal body fatness and CRC risk. The summary RR were 1.04 [95% (CI)1.02–1.05, *I*² = 91%] per 5 cm increase in height, 1.02 [95% (CI)1.01–1.02, *I*² = 0%] per 5 kg increase in weight, 1.06 [95% (CI)1.04–1.07, *I*² = 83%] per 5 kg/m^2^ increase in BMI, 1.02 [95% (CI)1.02–1.03, *I*² = 4%] per 10 cm increase in waist circumference, 1.03 [95% (CI)1.01–1.05, *I*² = 16%] per 0.1 unit increase in waist to hip ratio. The significant association for height and CRC risk was similar in men and women. The significant association for BMI and CRC risk was stronger in men than in women.

**Conclusion:**

The positive association between height and risk of CRC suggests that life factors during childhood and early adulthood might play a role in CRC aetiology. Higher general and abdominal body fatness during adulthood are risk factors of CRC and these associations are stronger in men than in women.

**Electronic supplementary material:**

The online version of this article (doi:10.1007/s00394-017-1557-1) contains supplementary material, which is available to authorized users.

## Introduction

Colorectal cancer (CRC) is the second most common cancer in women and the third most common cancer in men with 614,000 new cases diagnosed among women and 746,000 cases in men worldwide in 2012. It is a leading cause of cancer-related death, resulting in around 700,000 deaths worldwide [1].

In the World Cancer Research Fund (WCRF)/American Institute for Cancer Research report from 2011, it was stated that the evidence that greater adult attained height increases colorectal cancer risk was convincing, based on the results from eight studies [2]. Since then, ten additional large cohort studies have been published on height and colorectal cancer [3–11]. In addition, Mendelian Randomization studies have suggested a causal association between height and colorectal cancer [12, 13], however, one of these found an association only among women [12]. No previous meta-analyses have examined the shape of the dose–response relationship between height and colorectal cancer, and the most recent meta-analysis did not investigate whether the association persisted in subgroup analyses stratified by study characteristics.

Adiposity is also an established risk factor for colorectal cancer, both as measured by body mass index (BMI) and waist circumference and waist-to-hip ratio. Although many studies have investigated the association between BMI and colorectal cancer [8, 9, 14–40], fewer studies have been published on abdominal fat measures and colorectal cancer risk. Although the World Cancer Research Fund (WCRF)/Continuous Update Project (CUP) 2011 report [2] concluded that there was convincing evidence for an association between both general and abdominal fatness and colorectal cancer, the analyses for waist circumference and colorectal, colon and rectal cancer were based on only 3, 6 and 3 studies, respectively [24, 27, 41–46]. Previous meta-analyses [47, 48] have not investigated the shape of the dose–response relationship between BMI, waist circumference or waist-to-hip ratio and colorectal cancer [47, 48]. New studies, that could have been included in our meta-analysis, have been published on BMI (*n* = 24) [8, 9, 11, 14, 15, 17–24, 49–51], waist circumference (*n* = 13) [9, 16, 18, 22–24, 52, 53] and waist-to-hip ratio (*n* = 6) [9, 16, 18, 49, 53] and colorectal cancer since the WCRF/CUP 2011 report and for this reason we conducted an updated meta-analysis of the available evidence from prospective studies. We aimed to clarify the strength and shape of the dose–response relationship between height, weight, BMI, waist circumference, and waist-to-hip ratio and colorectal cancer risk and to clarify any potential differences by sex, and geographical location.

## Materials and methods

### Search strategy

PubMed, Embase, CABAbstracts, ISI Web of Science, BIOSIS, LILACS, Cochrane library, CINAHL, AMED, National Research Register and In Process Medline were searched for studies on anthropometric measures including BMI, height, weight, waist circumference and waist to hip ratio, and colorectal cancer risk up to December 2015. The specific search criteria and the review protocol can be found at: http://www.wcrf.org/int/research-we-fund/continuous-update-project-findings-reports/colorectal-bowel-cancer.

### Study selection

The search was restricted to cohort (prospective, retrospective, case–cohort or nested case–control studies) studies which investigated the link between anthropometric measures and colorectal cancer risk and reported estimates of the relative risk (RR) (e.g., hazard ratio, risk ratio or odds ratio) and 95% confidence intervals (CIs) for the exposures of interest (BMI, height, weight, waist circumference, and waist-to-hip ratio), total number of cases and person years of follow-up. If there were multiple publications from the same study, the newest publication which included the largest number of cases was selected.

### Data extraction

From each publication, the following data were extracted: first author’s last name, year of publication, the study name, period of follow-up, sample size, age, sex, number of cases, country where the study was conducted, assessment method of exposure (self-reported vs measured), anthropometric measures, their quantities and their associated RRs and 95% CIs, and variables used in adjustment in the analysis. The update search and data extraction from January 2010 up to November 2016 was conducted by three authors (LA, AR, JGS) and was checked for accuracy by two authors (TN, LA).

### Statistical methods

The associations between height, weight, BMI, waist circumference and waist-to-hip ratio and colorectal, colon and rectal cancer were investigated using random effect models to calculate the summary RRs and 95% CIs to take into account heterogeneity across studies [54]. Q and *I*² statistics were used to determine heterogeneity [55] and were explored in stratified analyses. Low proportion of heterogeneity across studies was defined by an *I* ^2^ < 30%, moderate proportion by an *I* ^2^ = 30–50%, and high proportion by an *I* ^2^ ≥ 50%.

Continuous risk estimates were used directly when available in the articles, and for studies that only reported categorical data dose–response associations and 95% CIs were derived using generalized least-squares for trend estimation [56], which required the RRs and CIs associated to at least three categories of anthropometric measures, and number of cases and non-cases or person years of follow-up per category to be available. If only the total number of cases or person years was reported in the articles, and the exposure was categorised in quantiles, the distribution of cases or person years was calculated by dividing the total number of cases or person years by the number of quantiles.

The mean or median values per category were used if provided in the articles, or the midpoint was calculated for studies that only reported a range by category. If the range of the highest or lowest category was open-ended, its width was assumed to be the same as the adjacent category. If the results were reported for men and women separately, they were combined using a fixed effects meta-analysis before being pooled with other studies.

Small-study effects, such as publication bias, were assessed using funnel plots and Egger’s test [57].

A potential nonlinear dose–response association between anthropometric measures and colorectal cancer risk was assessed by calculating restricted cubic splines for each study with more than three categories of exposure, using three fixed knots at 10, 50, and 90% through the total distribution of the reported measurements, and combined them using multivariate meta-analysis. Indication of non-linearity was tested using likelihood ratio test.

For all analyses, the results of each paper with the most comprehensive adjustment for confounders were included. A two-tailed *p* < 0.05 was considered statistically significant.

Stata version 12 software (StataCorp, College Station, TX, USA) was used.

## Results

**Fig. 1:**
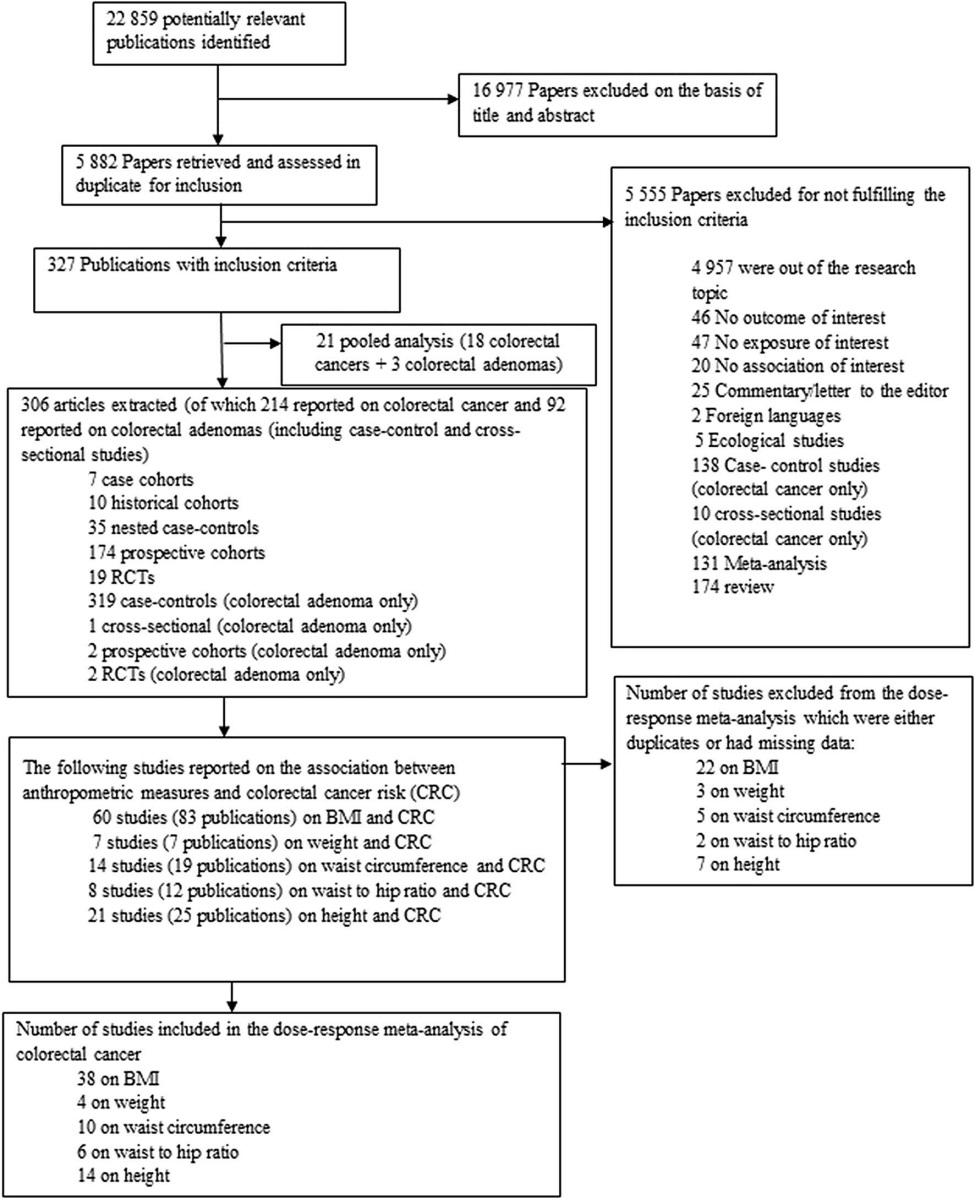
Flowchart of study selection

In total, 47 studies including 50,960 cases among 7,393,510 participants were included in the meta-analysis of anthropometric measures and colorectal, colon (proximal and distal), and rectal cancer risk (flowchart of study selection—Fig. 1). Characteristics of the included studies are provided in Supplemental Table 1.

### Height

#### Height and colorectal cancer

**Fig. 2:**
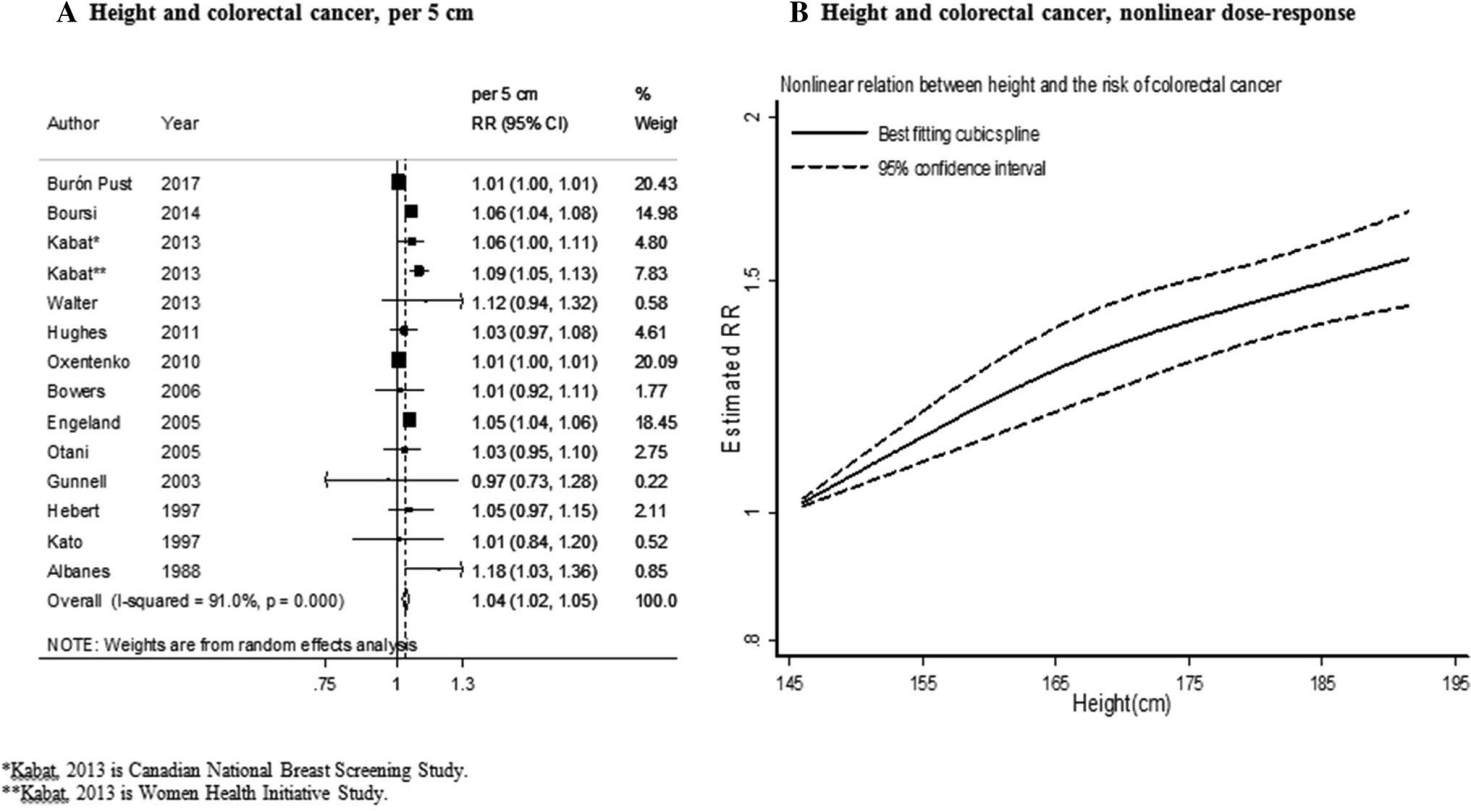
Height and colorectal cancer risk (dose–response and nonlinear analysis). *RR* relative risk, *95% CI* 95% confidence interval. Summary RR calculated using a random-effects model

Fourteen studies (84,095 cases) were included in the dose–response meta-analysis [3, 5, 6, 8, 9, 11, 26, 30, 58–63]. The summary RR for an increase of 5 cm was 1.04 (95% CI 1.02–1.05) (Fig. 2a). There was high heterogeneity (*I*² = 91%, *P*_heterogeneity_ < 0.001) and evidence of publication or small-study bias (*P* value Egger’s test < 0.05).

In stratified analysis the associations were similar in men and women and were stronger in studies in North America compared to studies in Europe. In addition, the associations tended to be stronger in studies in which height was measured than self-reported and in studies with less than 10 years of follow-up (Table 1).

**Table 1:** Summary of results

	Height, per 5 cm			
	*n*	RR (95% CI)	*I* ^2^ (%)	*P*_h_
Colorectal cancer				
All studies	14	1.04 (1.02–1.05)	91	< 0.001
Stratified by sex				
Men	8	1.04 (1.03–1.05)	0	0.46
Women	10	1.04 (1.02–1.05)	91.5	< 0.001
Stratified by geographic location				
Europe	6	1.03 (1.00–1.07)	95.6	< 0.001
North America	7	1.06 (1.01–1.11)	79.7	< 0.001
Asia	1	1.03 (0.95–1.10)	–	–
Australia	–	–	–	–
Duration of follow-up				
< 10 years follow-up	4	1.06 (1.04–1.08)	0	0.75
10–< 15 years follow-up	5	1.05 (1.00–1.11)	83.9	< 0.001
≥ 15 years follow-up	5	1.03 (1.00–1.07)	94.3	< 0.001
Assessment of height				
Measured	6	1.05 (1.04–1.06)	0	0.47
Self-reported	8	1.01 (1.00–1.02)	68.8	0.002
Number of cases				
Cases < 1000	7	1.05 (1.00–1.09)	0	0.58
Cases 1000–3000	4	1.04 (1.00–1.09)	87	< 0.001
Cases ≥ 3000	3	1.04 (1.00–1.08)	98	< 0.001
Colon cancer				
All studies	14	1.02 (1.02–1.03)	87	< 0.001
Stratified by sex				
Men	9	1.02 (1.01–1.04)	75.3	< 0.001
Women	12	1.02 (1.01–1.03)	85.7	< 0.001
Stratified by geographic location				
Europe	5	1.01 (1.00–1.02)	86	< 0.001
North America	6	1.05 (1.01–1.10)	89.7	< 0.001
Asia	2	1.12 (0.96–1.32)	79.2	0.03
Australia	1	1.13 (1.04–1.23)	–	–
Proximal colon cancer				
All studies	4	1.02 (0.99–1.05)	76.1	< 0.01
Stratified by sex				
Men	2	1.12 (0.90–1.40)	78.6	0.03
Women	4	1.01 (0.99–1.03)	52.3	0.09
Stratified by geographic location				
Europe	1	1.03 (0.96–1.11)	0	0.60
North America	2	1.01 (1.00–1.01)	0	0.82
Asia	–	–	–	–
Australia	1	1.24 (1.11–1.39)	0	0.74
Distal colon cancer				
All studies	4	1.01 (1.00–1.01)	0	0.85
Stratified by sex				
Men	2	1.05 (0.92–1.19)	45.5	0.17
Women	4	1.01 (1.00–1.02)	16.9	0.31
Stratified by geographic location				
Europe	1	1.05 (0.94–1.17)		
North America	2	1.01 (1.00–1.01)	0	0.58
Asia	–	–	–	–
Australia	1	1.04 (0.87–1.24)		
Rectal cancer				
All studies	14	1.01 (1.00–1.02)	61.7	0.002
Stratified by sex				
Men	10	1.02 (1.00–1.05)	39.7	0.09
Women	12	1.01 (1.00–1.01)	57.8	0.006
Stratified by geographic location				
Europe	6	1.00 (1.00–1.01)	37.0	0.16
North America	5	1.06 (1.02–1.10)	28.5	0.24
Asia	2	1.04 (1.00–1.09)	0	0.61
Australia	–	–	–	–

	Weight, per 5 kg			
	*n*	RR (95% CI)	*I* ^2^ (%)	*P*_h_
Colorectal cancer				
All studies	4	1.02 (1.01–1.02)	0	0.58
Stratified by sex				
Men	2	1.01 (1.00–1.02)	0	0.59
Women	1	1.02 (1.01–1.03)	–	–
Stratified by geographic location				
Europe	3	1.02 (1.01–1.03)	–	–
North America	1	1.02 (1.01–1.03)	–	–
Asia	–	–	–	–
Australia	–	–	–	–
Duration of follow-up				
< 10 years follow-up	1	1.02 (1.00–1.03)	–	–
10–< 15 years follow-up	1	1.01 (1.00–1.03)	–	^−^
≥ 15 years follow-up	2	1.02 (1.01–1.03)	5.7	0.30
Assessment of weight				
Measured	2	1.02 (0.99–1.05)	35.5	0.21
Self-reported	2	1.02 (1.01–1.03)	0	0.88
Number of cases				
Cases < 1000	3	1.02 (1.01–1.03)	0	0.43
Cases 1000–3000	–	–	–	–
Cases ≥ 3000	–	–	–	–
Colon cancer				
All studies	6	1.02 (1.01–1.03)	18	0.29
Stratified by sex				
Men	4	1.02 (1.01–1.02)	0	0.68
Women	2	1.05 (1.01–1.09)	6	0.30
Stratified by geographic location				
Europe	3	1.01 (1.00–1.02)	0	0.92
North America	1	1.03 (1.01–1.05)	–	–
Asia	–	–	–	–
Australia	2	1.05 (1.01–1.09)	6	0.30
Proximal colon cancer				
All studies	3	1.02 (1.00–1.04)	34	0.22
Stratified by sex				
Men	1	1.01 (0.98–1.04)		
Women	2	1.03 (0.98–1.09)	66.3	0.08
Stratified by geographic location				
Europe	1	1.01 (0.98–1.04)		
North America	1	1.01 (1.00–1.03)		
Asia	–	–	–	–
Australia	1	1.07 (1.01–1.14)		
Distal colon cancer				
All studies	3	1.03 (1.01–1.05)	23	0.27
Stratified by sex				
Men	1	1.02 (0.99–1.05)		
Women	2	1.05 (0.99–1.11)		
Stratified by geographic location				
Europe	1	1.02 (0.99–1.05)		
North America	1	1.02 (1.01–1.04)		
Asia	–	–	–	–
Australia	1	1.09 (1.01–1.18)		
Rectal cancer				
All studies	4	1.01 (1.00–1.02)	0	0.65
Stratified by sex				
Men	3	1.01 (1.00–1.02)	0	0.78
Women	1	1.06 (0.71–1.57)	–	–
Stratified by geographic location				
Europe	3	1.01 (1.00–1.02)	0	0.78
North America	1	1.04 (0.99–1.10)	–	–
Asia	–	–	–	–
Australia	–	–	–	–

	BMI, per 5 kg/m²			
	*n*	RR (95% CI)	*I* ^2^ (%)	*P*_h_^1^
Colorectal cancer				
All studies	38	1.06 (1.04–1.07)	83	< 0.001
Stratified by sex				
Men	20	1.08 (1.05–1.12)	83	< 0.001
Women	24	1.05 (1.03–1.07)	83.7	< 0.001
Stratified by geographic location				
Europe	10	1.04 (1.02–1.06)	80.8	< 0.001
North America	13	1.05 (1.03–1.07)	61.2	0.003
Asia	15	1.09 (1.01–1.18)	86.2	< 0.001
Australia	–	–	–	–
Duration of follow-up				
< 10 years follow-up	13	1.05 (1.03–1.07)	33.7	0.11
10–< 15 years follow-up	19	1.06 (1.03–1.09)	91.7	< 0.001
≥ 15 years follow-up	6	1.05 (1.02–1.07)	23.7	0.26
Assessment of weight/height				
Measured	25	1.05 (1.03–1.07)	28.1	0.17
Self-reported	13	1.06 (1.04–1.08)	85.9	< 0.001
Number of cases				
Cases < 1000	22	1.03 (1.02–1.05)	15.7	0.25
Cases 1000–3000	4	1.06 (1.03–1.09)	59.2	0.06
Cases ≥ 3000	11	1.09 (1.04–1.15)	97.6	< 0.001
Colon cancer				
All studies	42	1.07 (1.05–1.08)	78.8	< 0.001
Stratified by sex				
Men	26	1.10 (1.07–1.13)	74.2	< 0.001
Women	30	1.04 (1.02–1.05)	57	< 0.01
Stratified by geographic location				
Europe	13	1.05 (1.03–1.07)	80.6	< 0.001
North America	14	1.08 (1.05–1.11)	75.8	< 0.001
Asia	14	1.09 (1.03–1.16)	78.6	< 0.001
Australia	1	1.13 (1.00–1.28)	–	–
Proximal colon cancer				
All studies	20	1.05 (1.03–1.08)	44.0	0.04
Stratified by sex				
Men	12	1.13 (1.05–1.21)	33.2	0.20
Women	16	1.04 (1.01–1.07)	30.4	0.17
Stratified by geographic location				
Europe	1	1.12 (0.99–1.27)		
North America	11	1.04 (1.02–1.07)	33.1	0.13
Asia	8	1.16(1.06–1.27)		
Australia	–	–	–	–
Distal colon cancer			51.6	0.01
All studies	20	1.08 (1.04–1.11)		
Stratified by sex				
Men	12	1.23 (1.08–1.38)	77	< 0.01
Women	16	1.05 (1.03–1.08)	0	0.60
Stratified by geographic location				
Europe	1	1.34 (1.16–1.53)		
North America	11	1.05 (1.03–1.08)	0	0.53
Asia	8	1.18 (1.17–1.29)		
Australia	–	–	–	–
Rectal Cancer				
All studies	36	1.02 (1.01–1.03)	29.3	0.07
Stratified by sex				
Men	24	1.02 (1.01–1.04)	21.8	0.20
Women	25	1.01 (0.99–1.03)	43.6	0.02
Stratified by geographic location				
Europe	15	1.01 (1.00–1.02)	32.1	0.13
North America	11	1.02 (0.99–1.05)	23.9	0.24
Asia	14	1.04 (1.02–1.06)	0	0.71
Australia	1	1.02 (0.97–1.08)	–	–

	Waist circumference, per 10 cm			
	*n*	RR (95% CI)	*I* ^2^ (%)	*P*_h_^1^
Colorectal cancer				
All studies	10	1.02 (1.02–1.03)	4.2	0.40
Stratified by sex				
Men	5	1.03 (0.99–1.06)	77.9	0.001
Women	6	1.03 (1.02–1.04)	0	0.90
Stratified by geographic location				
Europe	2	1.01 (0.99–1.04)	34.4	0.22
North America	5	1.03 (1.01–1.04)	30.7	0.23
Asia	3	1.03 (1.01–1.05)	0	0.79
Australia	–	–		
Duration of follow-up				
< 10 years follow-up	5	1.02 (1.01–1.04)	0	0.97
10–<15 years follow-up	2	1.00 (0.98–1.03)	0	0.65
≥ 15 years follow-up	3	1.06 (0.98–1.14)	69.5	0.07
Assessment of waist circumference				
Measured	4	1.02 (1.00–1.05)	0	0.76
Self-reported	6	1.02 (1.01–1.04)	40.7	0.15
Number of cases				
Cases < 1000	9	1.02 (1.01–1.03)	12.9	0.33
Cases 1000–3000	1	1.03 (1.01–1.04)	–	–
Cases ≥ 3000	–	–		
Colon cancer				
All studies	11	1.05 (1.02–1.07)	72	< 0.001
Stratified by sex				
Men	7	1.09 (1.03–1.15)	95	< 0.001
Women	8	1.03 (1.01–1.04)	0.7	0.42
Stratified by geographic location				
Europe	2	1.02 (1.01–1.04)	0	0.88
North America	6	1.04 (1.01–1.08)	69.9	0.01
Asia	2	1.05 (1.02–1.08)	–	–
Australia	–	–	–	–
Proximal colon cancer				
All studies	5	1.05 (1.01–1.09)	49.9	0.09
Stratified by sex				
Men	2	1.12 (0.95–1.31)	79	0.03
Women	2	1.03 (1.01–1.05)	0	0.50
Stratified by geographic location				
Europe	1	1.04 (0.98–1.10)		
North America	4	1.06 (1.01–1.11)	62.1	0.05
Asia	–	–	–	–
Australia	–	–	–	–
Distal colon cancer				
All studies	5	1.06 (1.02–1.11)	50.5	0.09
Stratified by sex				
Men	2	1.14 (0.94–1.38)	83.5	0.01
Women	2	1.14 (1.01–1.06)	0	0.65
Stratified by geographic location				
Europe	1	1.05 (0.98–1.11)		
North America	4	1.07 (1.01–1.14)	62.8	0.04
Asia	–	–	–	–
Australia	–	–	–	–
Rectal cancer				
All studies	8	1.03 (1.00–1.05)	45.7	0.10
Stratified by sex				
Men	6	1.02 (0.98–1.06)	59.2	0.03
Women	5	1.04 (1.01–1.06)	0	0.48
Stratified by geographic location				
Europe	2	1.02 (1.00–1.05)	0	0.59
North America	2	1.07 (0.95–1.09)	82.7	0.02
Asia	1	1.00 (0.97–1.04)	–	–
Australia	1	1.12 (0.99–1.27)	–	–

	Waist-to-hip ratio, per 0.1 unit			
	*n*	RR (95% CI)	*I* ^2^ (%)	*P*_h_
Colorectal cancer				
All studies	6	1.03 (1.01–1.05)	15.7	0.31
Stratified by sex				
Men	2	1.18 (0.88–1.60)	81.8	0.02
Women	4	1.03 (1.01–1.04)	0	0.72
Stratified by geographic location				
Europe	–	–	–	–
North America	4	1.04 (1.00–1.08)	0	0.99
Asia	2	1.02 (0.98–1.06)	68	0.08
Australia	–	–	–	–
Duration of follow-up				
< 10 years follow-up	1	1.04 (1.00–1.08)	–	–
10–<15 years follow-up	1	1.02 (0.97–1.07)	–	–
≥ 15 years follow-up	4	1.06 (0.98–1.14)	52.5	0.12
Assessment of waist-to-hip ratio				
Measured	2	1.08 (0.95–1.22)	76.2	0.04
Self-reported	2	1.02 (0.99–1.04)	36.7	0.21
Number of cases				
Cases < 1000	5	1.04 (1.00–1.08)	30.5	0.23
Cases 1000–3000	1	1.03 (1.01–1.04)	–	–
Cases ≥ 3000	–	–	–	–
Colon cancer				
All studies	7	1.16 (1.05–1.28)	82.5	< 0.001
Stratified by sex				
Men	4	1.17 (1.05–1.30)	88.2	< 0.001
Women	5	1.07 (1.00–1.15)	60.8	0.04
Stratified by geographic location				
Europe	1	1.27 (1.13–1.43)	–	–
North America	3	1.14 (0.98–1.32)	78.5	0.01
Asia	2	1.05 (1.00–1.10)	0	0.46
Australia	1	1.47 (1.27–1.71)	–	–
Proximal colon cancer				
All studies	4	1.13 (1.00–1.28)	72.5	< 0.01
Stratified by sex				
Men	2	1.41 (1.17–1.72)	0	0.68
Women	3	1.03 (0.96–1.11)	43.2	0.17
Stratified by geographic location				
Europe	–	–	–	–
North America	3	1.06 (0.95–1.09)	71.9	0.03
Asia	–	–	–	–
Australia	1	1.36 (1.10–1.38)	0	0.49
Distal colon cancer				
All studies	4	1.17 (1.01–1.35)	74.6	< 0.01
Stratified by sex				
Men	2	1.50 (0.91–2.47)	82.9	0.01
Women	3	1.04 (1.02–1.06)	0	0.73
Stratified by geographic location				
Europe	–	–	–	–
North America	3	1.04 (1.02–1.06)	0	0.58
Asia	–	–	–	–
Australia	1	1.52 (0.92–1.50)	78.5	0.03
Rectal cancer				
All studies	6	1.04 (1.01–1.08)	22.1	0.26
Stratified by sex				
Men	4	1.05 (1.02–1.09)	0	0.62
Women	4	1.06 (0.98–1.15)	44.9	0.14
Stratified by geographic location				
Europe	1	1.04 (0.98–1.09)	–	–
North America	2	1.03 (0.98–1.09)	0	0.63
Asia	2	1.12 (0.98–1.28)	0	0.96
Australia	1	1.24 (1.02–1.51)	–	–

*RR* relative risk; *95% CI* 95% confidence interval

No evidence of nonlinear association was observed (*P*_nonlinearity_ = 0.33, *n* = 10) (Fig. 2b).

#### Height and colon cancer

Fourteen studies (92,069 cases) were included in the dose–response meta-analysis [4, 5, 7, 26, 30, 33, 41, 45, 58, 64–67]. The summary RR for an increase of 5 cm was 1.02 (95% CI 1.02–1.03). There was high heterogeneity (*I*² = 87%, *P*_heterogeneity_ < 0.001) (Table 1, Supplemental Fig. 1A). There was evidence of publication or small study bias (*P* value Egger’s test < 0.001).

In stratified analysis by sex and geographical location, the summary RR showed similar association in studies in men and women and stronger association in studies in North America than studies in Europe. No significant association was observed in studies in Asia including two studies (Table 1).

There was evidence of a significant non-linear association (*P*_nonlinearity_ = 0.03, *n* = 9), showing a significant increased risk with increasing height (Supplemental Fig. 1B).

#### Height and proximal and distal colon cancer

Four studies were included in the dose–response meta-analysis of height and proximal (1326 cases) and distal (1275 cases) colon cancer.

The summary RR for proximal colon cancer per an increase of 5 cm was 1.02 (95% CI 0.99–1.05) (Table 1 and Supplemental Fig. 2A). High heterogeneity was observed (*I*² = 76%, *P*_heterogeneity_ < 0.01). There was no evidence of publication bias (*P* value Egger’s test = 0.96).

In stratified analysis by sex, no significant association was observed. In stratified analysis by geographical location, a borderline significant increased risk was observed in studies conducted in North America.

There was no evidence of a non-linear association (*P*_nonlinearity_ = 0.41, *n* = 3) (Supplemental Fig. 2B).

The summary RR for distal colon cancer per an increase of 5 cm was 1.01 (95% CI 1.00–1.01) (Table 1; Supplemental Fig. 3A). No heterogeneity was observed (*I*² = 0%, *P*_heterogeneity_ = 0.85). There was a significant evidence of publication bias (*P* value Egger’s test = 0.04).

In stratified analysis by sex and geographical location, positive associations were borderline significant in studies in women and not in men, and in studies conducted in North America.

There was an evidence of a significant non-linear association (*P*_nonlinearity_ = 0.01, *n* = 3) (Supplemental Fig. 3B).

#### Height and rectal cancer

Fourteen studies (30,762 cases) were included in the dose–response meta-analysis [4, 5, 8, 11, 26, 30, 33, 41, 45, 58, 64–66]. The summary RR for an increase of 5 cm was 1.01 (95% CI 1.00–1.02) (Table 1; Supplemental Fig. 4A). There was high heterogeneity (*I*² = 62%, *P*_heterogeneity_ = 0.002). There was evidence of a significant publication or small study bias (*P* value Egger’s test < 0.001).

In stratified analysis, the summary RR showed a slightly stronger association in studies in men than in women and a stronger association in studies in North America than in studies in Asia and Europe (Table 1).

There was no evidence of a non-linear association (*P*_nonlinearity_ = 0.08, *n* = 9) (Supplemental Fig. 4B).

### Weight

#### Weight and colorectal cancer

**Fig. 3:**
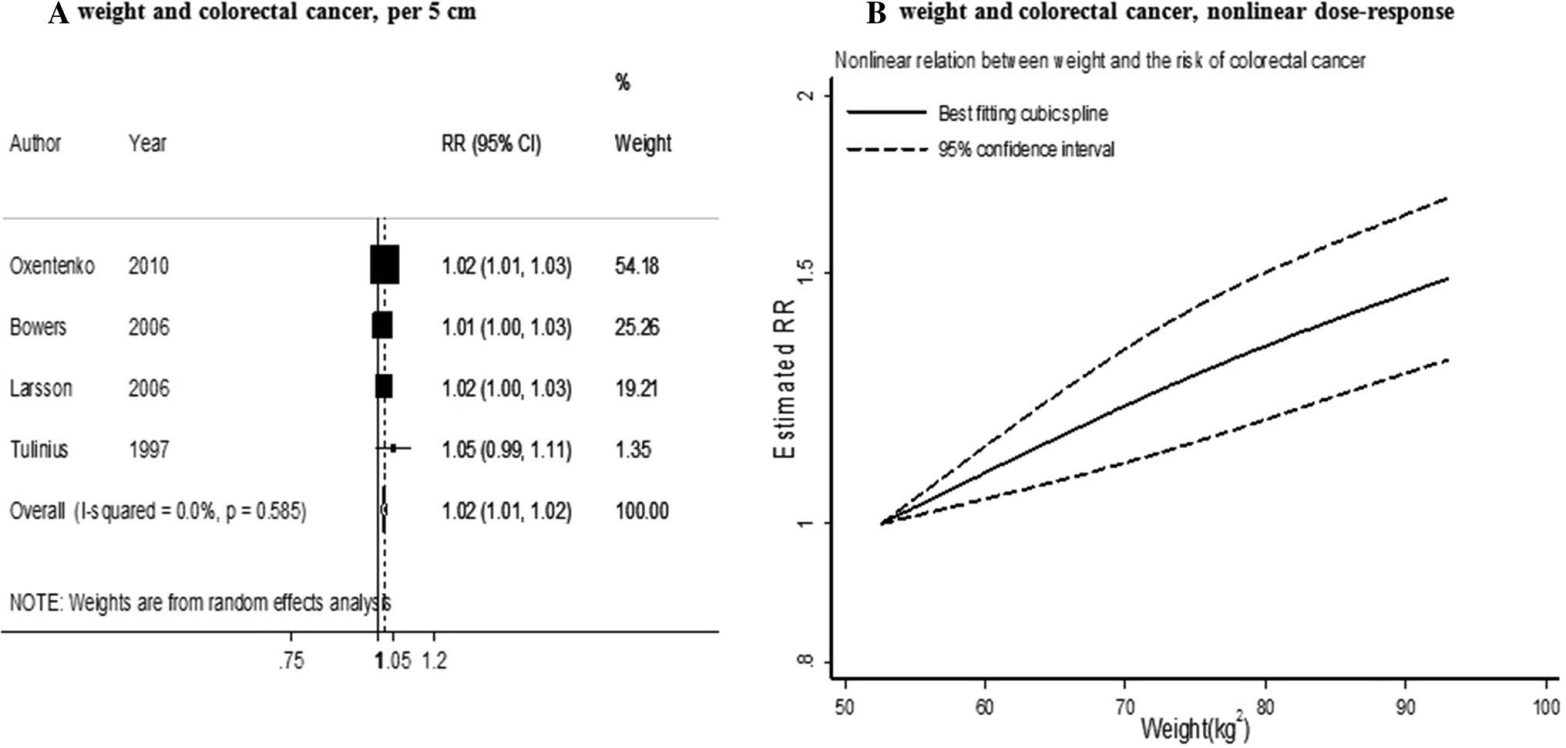
Weight and colorectal cancer risk (dose–response and nonlinear analysis). *RR* relative risk, *95% CI* 95% confidence interval. Summary RR calculated using a random-effects model

Four studies (2700 cases) were included in the meta-analysis [9, 26, 27, 39]. The summary RR per an increase of 5 kg was 1.02 (95% CI 1.01–1.02) (Fig. 3a) and there was no evidence.

of heterogeneity (*I*² = 0%, *P*_heterogeneity_ = 0.58). There was no evidence of a publication or small study bias (*P* value Egger’s test = 0.49).

In stratified analysis by sex and geographical location, positive significant associations were almost similar in both men and women and in studies conducted in Europe and North America.

There was no significant evidence of a non-linear association (*P*_nonlinearity_ = 0.43, *n* = 3) (Fig. 3b).

#### Weight and colon cancer

Six studies (2143 cases) were included in the meta-analysis [26, 27, 45, 68–70]. The summary RR per an increase of 5 kg was 1.02 (95% CI 1.01–1.03) (Table 1; Supplemental Fig. 5A). No heterogeneity was observed (*I*² = 18%, *P*_heterogeneity_ = 0.29). There was evidence of a significant publication or small study bias (*P* value Egger’s test = 0.01).

In stratified analysis, the summary RR showed stronger associations in studies in women than in men and in studies conducted in Australia than in Europe (Table 1).

There was no significant evidence of a non-linear association (*P*_nonlinearity_ = 0.84, *n* = 3) (Supplemental Fig. 5B).

#### Weight and proximal and distal colon cancer

Three studies were included in dose–response meta-analysis for proximal (1748 cases) and distal (1083 cases) colon cancer.

The summary RR for proximal colon cancer per an increase of 5 kg was 1.02 (95% CI 1.00–1.04) (Table 1; Supplemental Fig. 6A). No heterogeneity was observed (*I*² = 34%, *P*_heterogeneity_ = 0.22). There was no evidence of publication bias (*P* value Egger’s test = 0.73).

In stratified analysis by sex, no significant association was observed.

There was no significant evidence of a non-linear association (*P*_nonlinearity_ = 0.31, *n* = 2) (Supplemental Fig. 6B).

The summary RR for distal colon cancer per an increase of 5 cm was 1.03 (95% CI 1.01–1.05) (Table 1; Supplemental Fig. 7A). No heterogeneity was observed (*I*² = 23%, *P*_heterogeneity_ = 0.27). There was no evidence of publication bias (*P* value Egger’s test = 0.76).

In stratified analysis by sex, no significant association was observed.

There was no significant evidence of a non-linear association (*P*_nonlinearity_ = 0.48, *n* = 2) (Supplemental Fig. 7B).

#### Weight and rectal cancer

Four studies (1186 cases) were included in the meta-analysis [26, 27, 41, 45]. The summary RR per an increase of 5 kg was 1.01 (95% CI 1.00–1.02) (Table 1; Supplemental Fig. 8A). No heterogeneity was observed (*I*² = 0%, *P*_heterogeneity_ = 0.65) (Table 1).

In stratified analysis by geographical location, positive significant association was observed in studies in Europe.

There was no significant evidence of a non-linear association (*P*_nonlinearity_ = 0.29, *n* = 3) (Supplemental Fig. 8B).

### Body mass index (BMI)

#### BMI and colorectal cancer

**Fig. 4:**
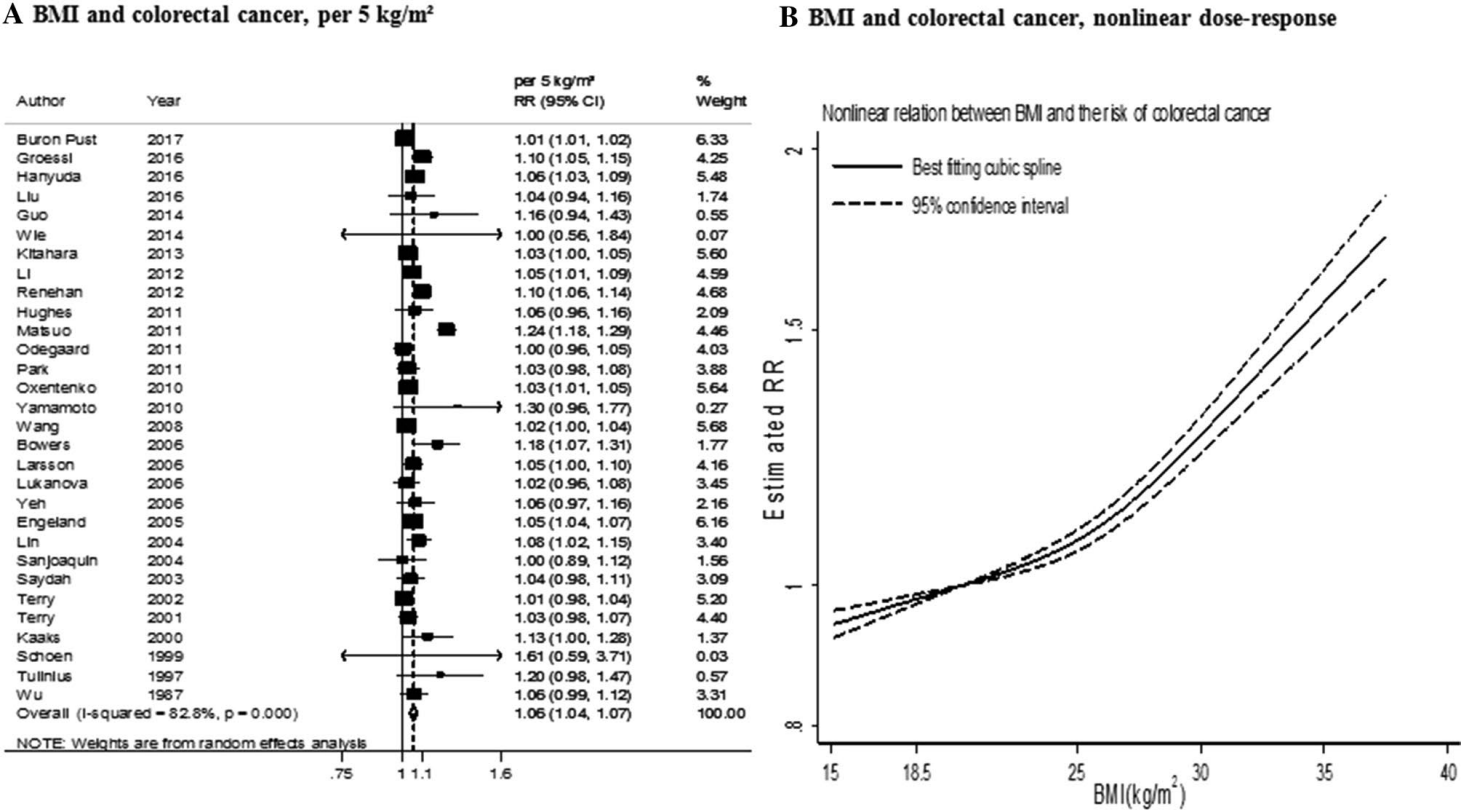
BMI and colorectal cancer risk (dose–response and nonlinear analysis). *RR* relative risk, *95% CI* 95% confidence interval. Summary RR calculated using a random-effects model

Thirty-eight studies (84 859 cases) were included in the dose–response meta-analysis [8, 9, 11, 14, 15, 18–24, 26–32, 34–40, 50, 51]. The summary RR for an increase of 5 kg/m^2^ was 1.06 (95% CI 1.04–1.07) (Fig. 4a). There was evidence of high heterogeneity (*I*² = 83%, *P*_heterogeneity_ < 0.001). There was evidence of a publication or small study bias (*P* value Egger’s test < 0.001).

Several differences in associations emerged in stratified analyses by study size, years of follow-up and in studies in which weight and height were self-assessed and in those in which they were measured, but none of them were statistically significant (Table 1). The associations tended to be stronger in men than in women and when the analysis was restricted to studies that reported in both sex, the summary RR were 1.05 (95% CI 1.03–1.07) in women and 1.08 (95% CI 1.05–1.12) in men. The association was stronger in studies in Asia than in studies conducted in Europe and North America. The associations tended to be stronger in studies in which weight and height were self-reported compared to measured, in studies with higher number of cases (> 3000), and in studies with 10–< 15 years of follow-up. However, none of these variables independently explained the heterogeneity.

There was statistical evidence of a non-linear relationship (*P*_nonlinearity_ < 0.001, *n* = 29) (Fig. 4b). Colorectal cancer risk increased with increasing BMI and the risk appeared to be stronger from a BMI increases around 27 kg/m^2^ and above.

#### BMI and colon cancer

Forty two studies (85,295 cases) were included in the dose–response meta-analysis [11, 14, 18–21, 24, 26–28, 30, 31, 33, 35–37, 44, 45, 49, 66, 69–83]. The summary RR for an increase of 5 kg/m^2^ was 1.07 (95% CI 1.05–1.08) (Table 1; Supplemental Fig. 9A). There was high heterogeneity (*I*² = 79%, *P*_heterogeneity_ < 0.001). There was evidence of publication or small study bias (*P* value Egger’s test < 0.001).

In analysis stratified by sex and geographical location, the summary RR showed stronger associations in studies in men than women and stronger associations in studies in Asia and North America than studies in Europe (Table 1).

There was no statistical evidence of a non-linear association (*P*_nonlinearity_ = 0.09, *n* = 33) (Supplemental Fig. 9B).

#### BMI and proximal and distal colon cancer

Twenty studies were included in dose–response meta-analysis for proximal (8437 cases) and distal (14,985 cases) colon cancer. There was evidence of significant positive association between BMI and both proximal and distal colon cancer risk, that was slightly stronger for distal than for proximal cancer.

The summary RR for proximal colon cancer per an increase of 5 kg/m^2^ was 1.05 (95% CI 1.03–1.08) (Table 1; Supplemental Fig. 10A). Medium heterogeneity was observed (*I*² = 44%, *P*_heterogeneity_ = 0.04). There was no evidence of publication bias (*P* value Egger’s test = 0.06).

In stratified analysis by sex and geographical location, positive significant associations were stronger in men than in women and in studies conducted in Asia than in North America.

There was no statistical evidence of a non-linear association (*P*_nonlinearity_ = 0.14, *n* = 8) (Supplemental Fig. 10B).

The summary RR for distal colon cancer for an increase of 5 kg/m^2^ was 1.08 (95% CI 1.04–1.11) (Table 1; Supplemental Fig. 11A). High heterogeneity was observed (*I*² = 52%, *P*_heterogeneity_ = 0.02). There was no evidence of publication bias (*P* value Egger’s test = 0.08).

In stratified analysis by sex and geographical location, positive significant associations were stronger in men than in women and in studies conducted in Asia than in Europe and North America.

There was no statistical evidence of a non-linear association (*P*_nonlinearity_ = 0.49, *n* = 8) (Supplemental Fig. 11B).

#### BMI and rectal cancer

Thirty-six studies (73,186 cases) were included in the dose–response meta-analysis [8, 11, 14, 17–21, 24, 26–28, 30, 31, 33, 35, 36, 41, 45, 49, 66, 70–72, 75, 76, 79, 80]. The summary RR for an increase of 5 kg/m^2^ was 1.02 (95% CI 1.01–1.03) (Table 1; Supplemental Fig. 12A). There was no evidence of heterogeneity (*I*² = 29.3%, *P*_heterogeneity_ = 0.07). No evidence of publication or small study bias was detected (*P* value Egger’s test = 0.08).

In stratified analysis, the summary RR was statistically significant in men but not in women, and in studies in Asia and not in other geographic locations (Table 1).

There was evidence of a non-linear association (*P*_nonlinearity_<0.001, *n* = 28) (Supplemental Fig. 12B). The curve shows that there is no evidence of association for BMI < 27.5 kg/m^2^, but increased risk for BMI values above this level.

### Waist circumference

#### Waist circumference and colorectal cancer

**Fig. 5:**
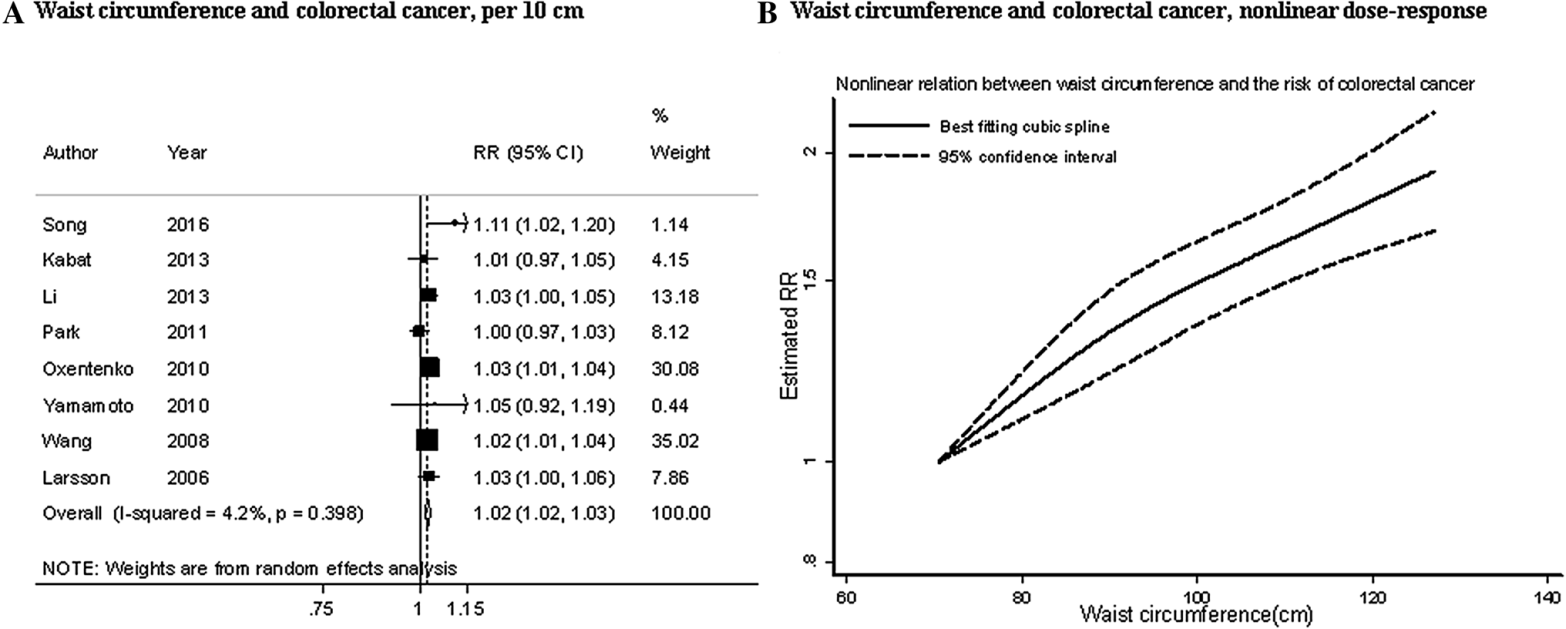
Waist circumference and colorectal cancer risk (dose–response analysis). *RR* relative risk, *95% CI* 95% confidence interval. Summary RR calculated using a random-effects model

Ten studies (1 884 cases) were included in the dose–response meta-analysis [9, 16, 18, 22–24, 27, 53]. The summary RR for an increase of 10 cm was 1.02 (95% CI 1.02–1.03) (Fig. 5a). There was no evidence of heterogeneity (*I*² = 4%, *P*_heterogeneity_ = 0.40) (Fig. 4a). There was no evidence of publication bias (*P* value Egger’s test = 0.60).

In stratified analysis, the positive associations were significant only in women and not in men, in studies conducted in Asia and North America and not in studies in Europe, and in studies with less years of follow-up (< 10 years). In studies with measured or self-reported waist circumferences, the positive association was similar (Table 1).

There was no evidence of non-linear association (*P*_nonlinearity_ = 0.17, *n* = 7) (Fig. 5b).

#### Waist circumference and colon cancer

Eleven studies (4729 cases) were included in the dose–response meta-analysis [16, 18, 24, 27, 42–45, 53, 68]. The summary RR for an increase of 10 cm was 1.05 (95% CI 1.02–1.07) (Table 1; Supplemental Fig. 13A). High heterogeneity was observed (*I*² = 72%, *P*_heterogeneity_ = < 0.001). There was evidence of publication bias (*P* value Egger’s test < 0.01).

In stratified analysis by sex and geographic location, the positive significant associations were stronger in men than in women and in studies in Asia and North America compared to the studies conducted in Europe (Table 1).

There was a significant evidence of non-linear association (*P*_nonlinearity_ = 0.001, *n* = 8). The curve shows increased risk of colon cancer with increasing waist circumference, with steeper associations at lower levels compared to higher levels of waist circumference (Supplemental Fig. 13B).

#### Waist circumference and proximal and distal colon cancer

Five studies were included in dose–response meta-analysis for proximal (1982 cases) and distal (1320 cases) colon cancer.

There were significant positive associations of similar magnitude between waist circumference and both proximal and distal colon cancer.

The summary RR for proximal colon cancer for an increase of 10 cm was 1.05 (95% CI 1.01–1.09). No heterogeneity was observed (*I*² = 49.9%, *P*_heterogeneity_ = 0.09) (Table 1; Supplemental Fig. 14A). There was no evidence of publication bias (*P* value Egger’s test = 0.41).

In stratified analysis by sex and geographical location, positive significant associations were observed in studies in women and not in men, and in studies conducted in North America.

There was no statistical evidence of a non-linear association (*P*_nonlinearity_ = 0.41, *n* = 3) (Supplemental Fig. 14B).

The summary RR for distal colon cancer per an increase of 10 cm was 1.06 (95% CI 1.02–1.11). No heterogeneity was observed (*I*² = 50.5%, *P*_heterogeneity_ = 0.09) (Table 1; Supplemental Fig. 15A). There was no evidence of publication bias (*P* value Egger’s test = 0.17).

In stratified analysis by sex and geographical location, positive significant associations were observed in studies in women and not in men, and in studies conducted in North America.

There was no statistical evidence of a non-linear association (*P*_nonlinearity_ = 0.84, *n* = 3) (Supplemental Fig. 15B).

#### Waist circumference and rectal cancer

Eight studies (1980 cases) were included in the dose–response meta-analysis of waist circumference and rectal cancer [18, 24, 27, 41, 45, 53]. The summary RR for an increase of 10 cm was 1.03 (95% CI 1.00–1.05) (Table 1; Supplemental Fig. 16A). No heterogeneity was observed (*I*² = 46%, *P*_heterogeneity_ = 0.10). There was no evidence of publication bias (*P* value Egger’s test = 0.30).

In stratified analysis by sex, significant association was observed only in women and not in men. In stratified analysis by geographic location, the positive association was borderline significant in studies in Europe and not significant in studies conducted in North America (Table 1).

There was no evidence of non-linear association (*P*_nonlinearity_ = 0.40, *n* = 6) (Supplemental Fig. 16B).

### Waist-to-hip ratio

#### Waist-to-hip ratio and colorectal cancer

**Fig. 6:**
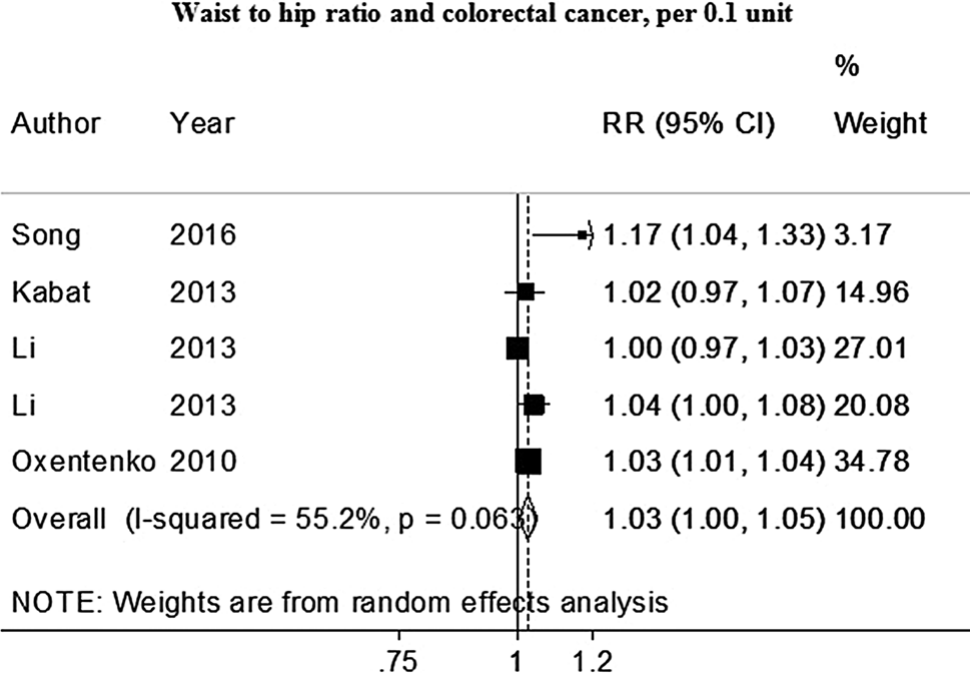
Waist-to-hip ratio and colorectal cancer risk (dose–response and nonlinear analysis). *RR* relative risk, *95% CI* 95% confidence interval. Summary RR calculated using a random-effects model

Six studies (4 689 cases) were included in the dose–response meta-analysis of waist to hip ratio and colorectal cancer [9, 16, 18, 49, 53]. The summary RR for an increase of 0.1 unit was 1.03 (95% CI 1.01–1.05) (Fig. 6).

No heterogeneity was observed (*I*² =16%, *P*_heterogeneity_ =0.31). There was no evidence of publication bias (*P* value Egger’s test = 0.436).

In stratified analysis, positive significant associations were observed only in studies in women and not in men, and in studies in North America and not in studies conducted in Asia (Table 1).

There was no statistical evidence of a non-linear association (*P*_nonlinearity_ = 0.34, *n* = 4) (Supplemental Fig. 6B).

#### Waist-to-hip ratio and colon cancer

Seven studies (3126 cases) were included in the dose–response meta-analysis of waist to hip ratio and colon cancer [18, 42, 43, 45, 49, 53, 68, 84]. The summary RR for an increase of 0.1 unit was 1.16 (95% CI 1.05–1.28) (Table 1; Supplemental Fig. 17A).

High heterogeneity was observed (*I*² =82%, *P*_heterogeneity_ < 0.001). There was no evidence of publication bias (*P* value Egger’s test = 0.14).

In stratified analysis by sex and geographic location, stronger association was observed in men compared to women and in studies in Europe than in studies in Asia (Table 1).

There was a significant evidence of non-linear association, showing colon cancer risk increased with increasing waist-to-hip-ratio (*P*_nonlinearity_ = 0.001, *n* = 7) and the curve was approximately linear across the range of waist-to-hip ratio values (Supplemental Fig. 17B).

#### Waist-to-hip ratio and proximal and distal colon cancer

Four studies were included in dose–response meta-analysis for proximal (1073 cases) and distal (932 cases) colon cancer. The summary RR for proximal colon cancer for an increase of 0.1 unit was 1.13 (1.00–1.28). High heterogeneity was observed (*I*² = 72.5%, *P*_heterogeneity_ <0.01) (Table 1; Supplemental Fig. 18). There was no evidence of publication bias (*P* value Egger’s test = 0.62).

In stratified analysis by sex, the positive association was only significant in men and not in women.

There was not enough studies to conduct non-linear analysis (*n* = 1).

The summary RR for distal colon cancer for an increase of 0.1 unit was 1.17 (95% CI 1.01–1.35). High heterogeneity was observed (*I*² = 74.6%, *P*_heterogeneity_ <0.01) (Table 1 and Supplemental Fig. 19). There was no evidence of publication bias (*P* value Egger’s test = 0.28).

In stratified analysis by sex and geographical location, the positive associations were significant only in women and not in men, and in studies in North America.

There was not enough studies to conduct non-linear analysis (*n* = 1).

#### Waist-to-hip ratio and rectal cancer

Six studies (1510cases) were included in the dose–response meta-analysis of waist to hip ratio and rectal cancer risk [18, 41, 45, 49, 53]. The summary RR for an increase of 0.1 unit was 1.04 (95% CI 1.01–1.08) (Table 1; Supplemental Fig. 20A). No heterogeneity was observed (*I*² =22%, *P*_heterogeneity_ =0.26). There was no evidence of publication bias (*P* value Egger’s test = 0.42).

In stratified analysis by sex, positive association was only significant in men and not in women. In stratified analysis by location, no significant association was observed (Table 1).

There was no statistical evidence of a non-linear association (*P*_nonlinearity_ = 0.14, *n* = 3) (Supplemental Fig. 20B).

## Discussion

In this meta-analysis of prospective studies, we quantified the evidence for the association between adult height, general obesity and abdominal fatness with CRC risk. To our knowledge this is the first dose–response meta-analysis to investigate the association between adult height and CRC risk. We found evidence of an increased risk of colorectal cancer with greater adult height. The association shows a 4% increased risk per an increment of 5 cm of height for colorectal, 2 and 1% increased risk for colon cancer and rectal cancer, respectively. The trend of increasing risk with greater adult height was similar in men and women, which is in contradiction with the results of several previous observational studies, showing stronger association among women than in men [8, 9, 12, 26, 30, 45, 65, 67, 68]. Also stronger association was observed in studies in which height was measured rather than self-reported. Moreover, there was an evidence of a non-linear association for greater adult height and colon cancer risk, with steeper associations at lower compared to higher levels of height.

The specific mechanism explaining the association between greater height and colorectal cancer risk has not been elucidated, although there is a great deal of evidences for an association between greater height and increased risk of other cancers including breast, pancreas, endometrium and ovarian cancers [85, 86]. Taller people have a greater number of cells in their body [87] and it has also been suggested that height is associated with the length of the intestines [88]; therefore, taller people might have a higher risk of cell mutations leading to malignancy. Elevated levels of insulin-like growth factor-1 (IGF-1) may play an important role in determining growth as higher IGF-1 levels in childhood are associated with childhood growth [34]. Elevated levels of IGF-1 may contribute to cancer risk by inhibiting apoptosis, stimulating cell proliferation and synthesis of sex steroids and inhibiting the synthesis of steroid hormone binding globulin [85, 89]. Nutritional status and diseases particularly infections during childhood and adolescence are play an important role in determining adult height [85, 90, 91] as poor nutrition and infections are the main reasons of growth failures in early childhood [92, 93]. Furthermore, it has been suggested that adult height represents the balance between nutritional intake and losses over time, specifically during the growth periods, including losses due to physical activity, psychological stress, and disease from the conception to maturity [92] and consequently adult height is a product of cumulative net nutrition [92]. Cancer risk in adulthood might be related to early life conditions but why these conditions might differentially affect men and women is unknown.

Our findings also show a positive relationship between all the included anthropometric factors (weight, BMI, waist circumference and waist to hip ratio) and the risk of colorectal cancer, and all the anatomical localizations (colon, proximal colon, distal colon, and rectal). Almost all positive associations were statistically significant.

The strongest association for BMI was observed with distal colon cancer, showing 8% increased risk for an increase of 5 kg/m² of BMI. The trend of increasing colorectal cancer risk with greater BMI was stronger in men than in women.

For waist circumference and waist to hip ratio, the strongest associations were observed with colon cancer, showing 5 and 16% increased risk per an increase of 10 cm of waist circumference and 0.1 increment of waist to hip ratio, respectively. Our results for an association between BMI and waist circumference and colorectal cancer risk are in agreement with the findings of two previous meta-analyses [47, 48].

We observed a nonlinear association between BMI and colorectal cancer risk. Colorectal cancer risk increased with BMI which appears to be stronger from BMI above 27 kg/m^2^ approximately. There was also evidence of non-linear association (*P* = 0.02) for BMI and colon cancer risk but the curve appeared approximately linear across the range of BMI values investigated. Furthermore, nonlinear associations were observed for abdominal fatness measures (waist circumference and waist to hip ratio) and colon cancer risk, with steeper associations at lower levels compared to higher levels of waist circumference. Several mechanisms are hypothesized to link obesity to colorectal cancer. Metabolic syndrome, elevated levels of insulin and insulin-like growth factors which inhibits apoptosis and modulates cell proliferation are suggested to play a role in the aetiology of colorectal cancer [48, 94–96]. There are evidences suggesting that abdominal fatness measured by high waist circumference and waist to hip ratio is a better indicator of metabolic disturbances, that affect the risk of colorectal cancer, than general obesity measured by high BMI as BMI has the limitation of not distinguishing between fat mass and lean mass [53].

This study has several advantages. It is based on large-scale prospective studies which minimize the probability of recall or selection bias, and also includes a large number of studies with relatively long follow-up and large number of cases that significantly increase the statistical power of the analysis. Moreover, the majority of the included studies in our meta-analysis were adjusted at least for age and other potential confounders such as alcohol consumption, smoking and physical activity.

However, the current meta-analysis has some limitations which should be taken into account when interpreting the results. High heterogeneity was observed across studies, which would affect the reliability of the summary RR estimates and lead to less accurate results. We did not find an explanation for this heterogeneity as it persisted in most subgroup analyses. This high heterogeneity might be due to variations and differences in anthropometric measurements categories. Another limitation of this meta-analysis can be due to measurement errors in the assessment of anthropometric measures, although most of the studies reported measured BMI (*n* = 27) rather than self-reported (*n* = 16).

In conclusion, our findings support the existing evidence of a positive association of general and abdominal body fatness with risk of CRC. In addition, higher adult height is significantly associated with increased colorectal cancer risk, particularly in women. These findings suggest that early life nutrition might play a role in colorectal cancer risk in adulthood.

## Electronic supplementary material

Below is the link to the electronic supplementary material.

Supplementary material 1 (PDF 939 KB)
