# Supplementary material for: Height and body fatness and colorectal cancer risk: an update of the WCRF–AICR systematic review of published prospective studies
Source: Eur J Nutr. 2017 Oct 28;57(5):1701–20. doi: 10.1007/s00394-017-1557-1 (PMC6060816; doi:10.1007/s00394-017-1557-1)

Supplementary Table 1. Main characteristics of included studies

Author, Year, Country	Study name, characteristics	Cases/ Study size Follow-up (years)	Case ascertainment	Exposure assessment	Exposure	Outcome	Adjustment factors
Burón Pust A, 2017 UK	Million Women Study, Prospective Cohort, Age: 56 years, W, Screening Program	18 518/ 1 310 390 13.8 years	Record linkage with the UK/NHS central register	Self-reported	BMI	Incidence, colorectal cancer	Age, age at menarche, age at menopause, alcohol, height, HRT use, hysterectomy, number of birth, oral contraceptive use, parity, region, smoking, socio-economic status, sterilisation, strenuous exercise
		12 761				Incidence, colon cancer	
		5 757				Incidence, rectal cancer	
		18 518/			Height	Incidence, colorectal cancer	
		12 761				Incidence, colon cancer	
		5 757				Incidence, rectal cancer	
Groessl, 2016 USA	WHI, Prospective Cohort, Age: 50-79 years, W, Postmenopausal	1 282/ 83 778 12.9 years	Self-report verified by pathology reports and medical records	Physical measurements such as height and weight were collected by trained staff at a baseline clinical visit.	BMI	Incidence, colorectal cancer	Age, alcohol, calcium, coffee, education, energy intake, ethnicity, family history of colorectal cancer, fibre, fruit and vegetables intake, history of diabetes, hormone use, NSAID use, pack years of smoking, percent calories from fat, period, physical activity, red meat intake
Hanyuda, 2016 USA	NHS-HPFS, Prospective Cohort, Age: 30-75 years, M/W, generally healthy	1 436/ 3 346 752 person-years	Self-report, death report, national death index, medical records reviewed by	In both cohorts, weight and height were self-reported at baseline	BMI	Incidence, colorectal cancer	Age, alcohol consumption, calcium intake, diet quality score, endoscopy, family history of colorectal cancer, folate, history of diabetes,

	adults		physicians				HRT use, multivitamin use, nsaid use, pack-years of cigarette smoking, physical activity, red and processed meat, regular aspirin use, total energy intake
Liu, 2016 China	SWHS, Prospective Cohort, Age: 40-70 years, W	870/ 68 253 15.1 years	Cancer registry, follow up by home visit, medical and hospital records	All participants were asked to wear lightweight indoor clothing and were measured for weight, height and circumferences of the waist and hips by trained interviewers, who were retired health professionals	BMI	Incidence, colorectal cancer	Alcohol consumption, educational level, family history of cancer, fruits and vegetables consumption, hormone replacement therapy, leisure time physical activity, meat intake, menopausal status, parity, total energy intake, spouse smoking exposure
		551				Incidence, colon cancer	
		319				Incidence, rectal cancer	
		870			Waist-to-hip ratio	Incidence, colorectal cancer	
		551				Incidence, colon cancer	
		319				Incidence, rectal cancer	
Song, 2016 USA	NHS-HPFS, Prospective Cohort, Age: 30-75 years, M/W, Health professionals	1 125/ 112 610 1 979 428 person-years	Self-report, next of kin, medical and pathological records	Self-reported via questionnaire	BMI	Incidence, colorectal cancer	Age, alcohol, calcium intake, calendar year, diet quality, endoscopy, family history of colorectal cancer, height, multivitamin, NSAID use, pack-years of smoking, physical activity
		1 884			Waist-circumference	Incidence, colorectal cancer	
		1 365				Incidence, colon cancer	
		401				Incidence, rectal cancer	
		1 877			Waist-to-hip ratio	Incidence, colorectal cancer	
		1 361				Incidence, colon cancer	
		399				Incidence, rectal cancer	
Bhaskaran, 2014	CPRD,	13 465/	Medical records	Weight and height	BMI	Incidence,	Age, sex, diabetes, socio-

UK	Prospective Cohort,<br>Age: 16- years,<br>M/W	5 243 978<br>25 years		were measured		colon cancer	economic status, alcohol, calendar year
		6 123/				Incidence,<br>rectal cancer	
Guo, 2014<br>China	Northern China 2006-2011,<br>Prospective Cohort,<br>Age: 18- years,<br>M/W	149/<br>133 273<br>4.28 years	Self-report, next of kin, medical and pathological records	Weight and height were measured	BMI	Incidence,<br>colorectal cancer	Age, alcohol consumption, education level, smoking
		62/				Incidence,<br>colon cancer	
		86/				Incidence,<br>rectal cancer	
Wie, 2014<br>Korea	Korea 2004-2008,<br>Prospective Cohort,<br>M/W	53/<br>8024<br>7 years	Cancer registry and medical records	Weight and height were measured	BMI	Incidence,<br>colorectal cancer	Age, sex, energy intake, smoking, physical activity, alcohol use, income, education and marital status
Song, 2014<br>Finland	FINRISK,<br>Prospective Cohort,<br>Age: 24-74 years,<br>M/W	203/<br>54 725<br>20.6 years	Cancer and mortality registries	Height and weight were measured on site by specially trained nurses	BMI	Incidence<br>colon cancer, men	Age, leisure - physical activity, area, education, smoking
		184/				Colon, women	
		203/					
		184/				Incidence<br>rectum, men	
		103/				Incidence<br>rectum, women	
Boursi, 2014<br>UK	THIN,<br>Nested Case Control,<br>Age: 40- years,<br>M/W	5 617/<br>15122 controls	Medical records		Height	Incidence,<br>colorectal cancer, men	Alcohol, BMI, colonoscopy, connective tissues disease, diabetes, heart disease, NSAID use, smoking
		4 361/<br>11725 controls				Incidence,<br>colorectal cancer, women	
Kabat, 2014<br>USA	NIH-AARP,<br>Prospective Cohort,<br>Age: 50-71 years,<br>M/W,<br>Retired	1 311/<br>481 197<br>10.5 years	Cancer registry and national death Index	Self-reported height	Height	Incidence,<br>colon cancer, women	Age, age at menarche, alcohol, BMI, colonoscopy, educational level, family history of cancer, physical activity, race, smoking
		2 860/				Incidence,<br>colon cancer, men	
		1 427/				Incidence,<br>rectal cancer,	

						men	
		591/				Incidence, rectal cancer, women	
Kabat, 2013 USA	WHI, Prospective Cohort, Age: 50-79 years, W, Post-menopausal	166/ 11 124 12.9 years	Self-report verified by reviewing medical and pathological records by physicians	Weight, height, and waist and hip circumferences measured by trained staff	BMI<br><br>Waist-circumference<br><br>Waist to hip ratio	Incidence, colorectal cancer	Diabetes, ethnicity, HRT use, physical activity, alcohol, education, family history colorectal cancer, pack years smoking randomisation group
		1 516/ 144 701 12 years			Height		
		1 904/ 144 701 12 years				Incidence, colon cancer	
		257/ 481 197 10.5 years				Incidence, rectal cancer	
Kitahara, 2013 USA	PLCO, Prospective Cohort, Age: 55-74 years, M/W	962/ 74 474 11.9 years	Self-reported/death certificate/ medical records	Questionnaire	BMI	Incidence, colorectal cancer, all	Sex, HRT use, race, study centre, age at baseline, screening, smoking
		546/				Colorectal, men	
		416/				Colorectal, women	
		529/				Proximal colon, all	
		275/				Proximal colon, men	
		254/				Proximal colon, women	
		219/				Distal colon, all	

		131/				Distal colon, men	
		85/				Distal colon, women	
		200/				Rectum, all	
		134/				Rectum, men	
		66/				Rectum, women	
Morikawa, 2013<br><br>USA	NHS & HPFS, Pooled analysis, (W) nurses and (M) health professionals	861/<br>156 703<br>2 631 423 person-years	Self-report (provided evidence of treatment), medical records and pathology reports, national death Index	Weight measured, height was self-reported in 1976	BMI	Incidence, colorectal cancer	Age, aspirin, calcium, caloric intake, folate, red meat, vit D, multivitamin, smoking status, alcohol, family history CRC, sigmoidoscopy, physical activity smoking, HRT use (women)
	NHS, Prospective Cohort, W	493/<br>109 046					
	HPFS, Prospective Cohort, M	368/<br>47 684					
Poynter, 2013<br>USA	IWHS, Prospective Cohort, Age: 55-71 years, W, Post-menopausal	707/<br>37 459<br>22 years	SEER	BMI was calculated from self-reported current weight and height	BMI	Incidence, colon cancer, aged ≥75 years	Estrogen use, physical activity, age at baseline, smoking
		604/				Incidence, colon cancer, aged < 75 years	
Kabat, 2013<br>Canada	CNBSS, Prospective Cohort, Age: 40-59 years, W	1 096/<br>88 256<br>16.2 years	Record linkages to cancer database and to the national mortality database	Height and weight measured at the Initial examination.	Height	Incidence, colorectal cancer	Age, BMI, hormone replacement therapy, menopausal status, oral contraceptive use, pack years of smoking, years of education
		769/				Incidence, colon cancer	
		338/				Incidence, rectal cancer	
Walter, 2013<br>USA	VITAL, Prospective Cohort, Age: 50-76 years, M/W	491/<br>65 038<br>7.3 years	Cancer registry	Questionnaire	Height	Incidence, colorectal cancer	Age, sex, race
Morikawa, 2013<br><br>USA	NHS & HPFS, Pooled analysis, (W) nurses and (M) health	861/<br>156 703<br>2 631 423 person-	Self-report (provided evidence of treatment), medical records and	Weight measured, height was self-reported in 1976	BMI	Incidence, colorectal cancer	Age, aspirin, calcium, caloric intake, folate, red meat, vit D, multivitamin, smoking status, alcohol, family history CRC,

	professionals	years	pathology reports, national death Index				sigmoidoscopy, physical activity smoking, HRT use (women)
	NHS, Prospective Cohort, W	493/ 109 046					
	HPFS, Prospective Cohort, M	368/ 47 684					
Li, 2012 China	SWHS, Prospective Cohort, Age: 40-70 years, W	621/ 72 972 11 years	Follow up survey/cancer registry/vital statistics registry	Anthropometric measurements, measured by trained Interviewers at baseline	BMI<br><br>Waist-circumference<br><br>Waist to hip ratio	Incidence, colorectal cancer	Alcohol consumption, energy intake, fruit, Income, menopausal status, physical activity, tea consumption, age at baseline, cigarette, education, family history of colorectal cancer, red meat, vegetables
		381/				Incidence, colon cancer	
		240/				Incidence, rectal cancer	
Li, 2012 China	SMHS, Prospective Cohort, Age: 40-74 years, M	313/ 61 283 5.5 years	Follow up survey/cancer registry/vital statistics registry	Anthropometric measurements, measured by trained Interviewers at baseline	BMI<br><br>Waist-circumference<br><br>Waist to hip ratio	Incidence, colorectal cancer	Alcohol consumption, energy intake, fruits, Income, physical activity, tea consumption, age at baseline, education, family history of colorectal cancer, pack years of smoking, red meat, vegetables
		180/				Incidence, colon cancer	
		133/				Incidence, rectal cancer	
Renehan, 2012 USA	NIH-AARP, Prospective Cohort, Age: 50-71 years, M/W	4 076/ 273 679 2 509 662 person years	Cancer registry	Assessed by questionnaire, recalled weights at ages 18, 35, and 50 years and self-reported height	BMI	Incidence, colorectal cancer, men	Age, physical activity, race, alcohol, education, smoking, alcohol consumption, HRT use (in women)
		2 804/					
		1 240/				Incidence, colorectal cancer, women	
		2 070/				Incidence, colon cancer, men	
		1 145/				Incidence, proximal colon cancer, men	

		855/				Incidence, distal colon cancer, men	
		962/				Incidence, colon cancer, women	
		607/				Incidence, proximal colon cancer, women	
		329/				Incidence, distal colon cancer, women	
		762/				Incidence, rectal cancer, men	
		282/				Incidence, rectal cancer, women	
Hughes, 2011 Netherlands	NLCS, Prospective Cohort, Age: 55-69 years, M/W	1 211/ 120 852 16.3 years	Cancer registry	Self-reported height and weight	BMI	Incidence, colorectal cancer, men	Age, alcohol consumption, energy intake, occupational activity, education, family history of colorectal cancer, smoking
		1 211/					
		1 106/				Incidence, colorectal cancer, women	
		327/				Incidence, proximal colon cancer, men	
		427				Incidence, distal colon cancer, men	
		459/				Incidence, proximal colon cancer, women	
		327/				Incidence, distal colon cancer, women	

		299/				Incidence, rectal cancer, men	
		205/				Incidence, rectal cancer, women	
Odegaard, 2011 Singapore	SCHS, Prospective Cohort, Age: 45-74 years, M/W, middle-aged adults	980/ 51 251 11.5 years	Cancer registry and death registry	Self-reported height and weight	BMI	Incidence, colorectal cancer	Age, sex, diabetes, dialect group, dietary pattern score, family history of cancer, physical activity, sleep, alcohol intake, education, energy, smoking, year
		596/				Incidence, colon cancer	
		384/				Incidence, rectal cancer	
Park, 2011 UK	EPIC-Norfolk, Prospective Cohort, Age: 40-79 years, M/W	357/ 20 608 11 years	Record linkage to cancer registration and death certificates	Self-reported and measured	BMI<br><br>Waist-circumference	Incidence, colorectal cancer, men (measured BMI)	Age, sex, smoking, alcohol, education, exercise, family history of CRC, energy intake, folate, fibre, total meat and processed meat, intakes
						Incidence, colorectal cancer, men (self-reported BMI)	
						Incidence, colorectal cancer, women (measured BMI)	
						Incidence, colorectal cancer, women (self-reported BMI)	
						Men (measured BMI)	Age, sex, s moking, alcohol, education, exercise, family history of CRC, energy intake, folate,

						Men (self-reported BMI)	fibre, total meat and processed meat, intakes, waist and hip circumferences
						Women (measured BMI)	
						Women (self-reported BMI)	
Odegaard, 2011 Singapore	SCHS, Prospective Cohort, Age: 45-74 years, M/W, middle-aged adults	980/ 51 251 11.5 years	Cancer registry and death registry	Self-reported height and weight	BMI	Incidence, colorectal cancer	Age, sex, diabetes, dialect group, dietary pattern score, family history of cancer, physical activity, sleep, alcohol intake, education, energy, smoking, year
		596/				Incidence, colon cancer	
		384/				Incidence, rectal cancer	
		589/				Incidence, colorectal cancer, never smokers	
						Ever smokers	
		391/				Incidence, colon cancer, never smokers	
		205/				Incidence, colon cancer, ever smokers	
		198/				Incidence, rectal cancer, never smokers	
		176/				Ever smokers	
Matsuo, 2011 Japan	Pooling analysis of 8 cohort studies in Japan	3 055			BMI	Incidence, colorectal cancer, men	
		1 924				Incidence, colorectal cancer, women	
		1 919				Incidence, colon cancer,	

						men	
		1 534				Incidence, colon cancer, women	
		1 111				Incidence, rectal cancer, men	
		735				Incidence, rectal cancer, women	
Green, 2011<br>UK	MWS,<br>Prospective Cohort,<br>Age: 56 years,<br>W	6 281/<br>1 297 124<br>9.4 years	Cancer registry	Questionnaire	Height	Incidence, colon cancer	Age, age at first child birth, age at menarche, alcohol, BMI, parity, region, smoking, socio-economic status, strenuous exercise
Hughes, 2011<br>Netherlands	NLCS,<br>Prospective Cohort,<br>Age: 55-69 years,<br>M/W	1 211/<br>120 852<br>16.3 years	Cancer registry	Self-reported height and	Height	Incidence, colorectal cancer, men	Age, alcohol consumption, educational level, energy intake, family history of colorectal cancer, occupational activity, smoking, weight
		1 211/				Incidence, colorectal cancer, men	
		1 106/				Incidence, colorectal cancer, women	
		1 106/				Incidence, colorectal cancer, women	
		459/				Incidence, proximal colon cancer, women	
		459/				Incidence, proximal colon cancer, women	
		427/				Incidence, distal colon cancer, men	
		427/				Incidence, distal colon cancer, men	
		327/				Incidence, proximal colon	

						cancer, men	
		327/				Incidence, proximal colon cancer, men	
		327/				Incidence, distal colon cancer, women	
		327/				Incidence, distal colon cancer, women	
Oxentenko, 2010 USA	IWHS, Prospective Cohort, Age: 55-69 years, W	1 464/ 36 941 619 961 person-years	SEER	Self-reported height and weight at baseline	BMI<br><br>Waist-circumference<br><br>Waist to hip ratio<br><br>Weight	Incidence, colorectal cancer	Age, age at menopause, calcium, contraception, diabetes, energy intake, estrogen use, folate, smoking status, total fat, vitamin E, alcohol, cigarette consumption, fruit and vegetable, physical activity level, red meat
		771/				Incidence, proximal colorectal cancer	
		660/				Incidence, distal colorectal cancer	
		1 464/ 36 941 619 961 person-years			Height	Incidence, colorectal cancer	
Yamamoto, 2010 Japan	HHCCS, Nested case-control study, Age: 54 years, M/W	22 cases/ 69 controls 3 years	Histology	Measured height and weight	BMI<br><br>Waist-circumference	Incidence, colorectal cancer	Age, sex, alcohol consumption, smoking status, year of examination
Bassett, 2010 Australia	MCCS, Prospective Cohort, Age: 40-69 years, M/W	569/ 41 154 14 years	Cancer registry	Self-reported	BMI<br><br>Weight	Incidence, colon cancer, women	Country of birth, energy intake, fat intake, fruit and vegetable consumption, education, processed and red meat, smoking
		292/					
		277/				Incidence, colon cancer, men	
Laake, 2010	NCS,	450/	Cancer registry	Height and weight	BMI	Incidence,	Age, area of residence,

Norway	Prospective Cohort,<br>Age: 20-49 years,<br>M/W,<br>Screening Program	76 179<br>23.2 years		was measured at examinations up to three times between 1974 and 1988		colon cancer, men	educational level, energy intake, height, physical activity, smoking status
		419/				Incidence, colon cancer, women	
		228/				Incidence, proximal colon cancer, men	
		174/				Incidence, distal colon cancer, men	
		237/				Incidence, proximal colon cancer, women	
		159/				Incidence, distal colon cancer, women	
Prentice, 2009<br>USA	WHI<br>Prospective Cohort,<br>Age: 50-79 years,<br>W,<br>postmenopausal women	363/<br>80 816<br>11 years	Self-report, medical record and pathology report reviewed by centrally trained physician		BMI	Incidence, colon cancer	Age, family history of colon cancer, physical activity, smoking status, alcohol, total energy intake
		87/				Incidence, rectal cancer	
Sung, 2009<br>Korea	KNHIC,<br>Prospective Cohort,<br>Age: 40-64 years,<br>M/W,<br>middle-class adults	2 499/<br>788 789<br>8.72 years	Cancer registry and death records	Weight and height were measured	Height	Incidence, colon cancer, men	Age, alcohol consumption, area of residence, BMI, occupation, physical activity, salary, smoking habits
		1007/				Incidence, colon cancer, women	
		2281/				Incidence, rectal cancer, men	
		892/				Incidence, rectal cancer, women	
Jee, 2008<br>Korea	KNHIC,<br>Prospective Cohort,<br>Age: 30-95 years,<br>M/W	4 671/<br>1 213 829<br>10.8 years	National cancer registries, hospitalisation records and admission files	Weight and height measurements were recorded during health examination at hospital	BMI	Incidence, colon cancer, men	Age, smoking status
		4 032/				Incidence, rectum cancer, men	

		1 959/				Incidence, colon cancer, women	
		1 681/				Incidence, rectum cancer, women	
Song, 2008<br>Korea	KNHIC,<br>Prospective Cohort,<br>Age: 40-64 years,<br>W,<br>Post-menopausal	453/<br>170 481<br>8.75 years	Self-report, cancer registry, death report	Weights and heights were measured	BMI	Incidence, colon cancer	Age, alcohol, height, physical activity, smoking status, pay level at study entry
Thygesen, 2008<br>USA	HPFS,<br>Prospective Cohort,<br>Age: 40-75 years,<br>M	693/<br>46 349<br>18 years	Self-report verified by medical record	Self-reported data	BMI	Incidence, colon cancer	Age, alcohol consumption, aspirin use, intakes of calcium, energy, folate, methionine, and multivitamin, physical activity, previous endoscopic screening, processed meat, red meat intake, smoking habits, vitamin d, family history of colorectal cancer
Wang, 2008<br>USA	CPS II,<br>Prospective Cohort,<br>M/W	546/<br>95 151<br>7.7 years	Self-report,<br>pathology report,<br>national death Index, death cert,<br>state cancer registries	Self-reported data	BMI<br><br>Waist-circumference	Incidence, colorectal cancer, men	Age, alcohol, educational level, height, history of endoscopy, multivitamin use, NSAID use, physical activity, smoking status
		407/				Incidence, colorectal cancer, Women	HRT use, Waist-circumference
		402/				Incidence, colon cancer, men	
		314/				Incidence, colon cancer, women	
		142/				Incidence, rectal cancer, men	
		93/				Incidence, rectal cancer, women	
Reeves, 2007<br>UK	MWS,<br>Prospective Cohort,	4 008/<br>1 222 630	National health records	Self-reported	BMI	Incidence, colorectal	Age, alcohol consumption, geographical area, physical

	Age: 50-64 years, W	5 years				cancer	activity, reproductive factors, smoking status, socio-economic status
		1 884/				Never smoked	
		1 743/				Postmenopausal never users of HRT	
		136/				Incidence, colorectal cancer, premenopausal never users of HRT	
Bowers, 2006 Finland	ATBC, Prospective Cohort, Age: 58 years, M, Smokers	410/ 28 983 14.1 years	Cancer registry	Measured by trained staff	BMI<br><br>Weight	Incidence, colorectal cancer	Age, no of cigarettes smoked
		227/				Incidence, colon cancer	
		183/				Incidence, rectal cancer	
		410/ 28 983 14.1 years			Height	Incidence, colorectal cancer	Age, number of cigarettes smoked, weight
		227/				Incidence, colon cancer	
		183/				Incidence, rectal cancer	
Larsson, 2006 Sweden	COSM, Prospective Cohort, Age: 45-79 years, M	461/ 45 906 7.1 years	Cancer registry	Self-reported height and weight at age 20, weight and waist circum. at baseline	BMI<br><br>Waist-circumference<br><br>Weight	Incidence, colorectal cancer	Age, aspirin use, BMI, educational level, history of diabetes, recreational activity, smoking status, family history of colorectal cancer
		284/				Incidence, colon cancer	
		180/				Incidence, rectum cancer	
		129/				Incidence, distal colon cancer	
		120/				Incidence,	

						proximal colon cancer	
Lukanova, 2006 Sweden	NSHDC, Prospective Cohort, Age: 29-61 years, M/W	136/ 68 786 8.2 years	Medical records	Weight and height measured by nurse	BMI	Incidence, colorectal cancer, men	Age, smoking habits, calendar year
		108/				Women	
		76/				Incidence, colon cancer, women	
		73/				Men	
		58/				Incidence, rectal cancer, men	
		31/				Women	
MacInnis, 2006 Australia	MCCS, Prospective Cohort, Age: 27-75 years, W	117/ 24 072 10.4 years	Cancer registry	Height, weight, waist and hip were measured	BMI<br><br>Waist-circumference<br><br>Weight	Incidence, proximal colon cancer	Age-underlying cox models, country of birth, educational level, HRT use
		79/				Incidence, distal colon cancer	
		212/ 24 072 10.4 years			Height	Incidence, colon cancer	
MacInnis, 2006 Australia	MCCS, Prospective Cohort, Age: 27-75 years, M/W	229/ 41 114 10.3 person-years	Cancer registry	Measured	BMI<br><br>Weight	Incidence, rectal cancer	Age, sex, country of birth
		134/				Incidence, rectal cancer , men	
		120/				Incidence, stage I/II rectal cancer	
		102/				Incidence, stage III/iv rectal cancer	

		95/				Incidence, rectal cancer, women	
		134/			Height	Incidence, rectal cancer	
		95/				Incidence, rectal cancer, men	
Pischon, 2006 Europe	EPIC, Prospective Cohort, Age: 25-70 years, M/W	563/ 368 277 2 254 727 person-years	Population registries	Self-reported questionnaires	BMI<br><br>Waist-circumference<br><br>Waist to hip ratio	Incidence, colon cancer, women	Alcohol consumption, centre, educational level, physical activity, smoking status, age at recruitment, fibre, fish and shellfish, fruits and vegetables, red and processed meat
		421/				Incidence, colon cancer, men	
		295/				Incidence, rectal cancer, men	
		291/				Incidence, rectal cancer, women	
		421/ 368 277 2 254 727 person-years			Height	Incidence, colon cancer, women	
		563/				Incidence, colon cancer, men	
		295/				Incidence, rectal cancer, men	
		291/				Incidence, rectal cancer, women	
Samanic, 2006 Sweden	SFOSHCIC, Historical Cohort,	1 795/ 362 552	Health screening programme	From health records	BMI	Incidence, colon cancer	Age, calendar year, smoking status

	Age: 18-67 years, M	19 years					
		1 362/				Incidence, rectal cancer	
		379/				Never smokers	
Yeh, 2006 Taiwan	Taiwan cohort, Prospective Cohort, Age: 30-65 years, M/W	68/ 23 943 10 years	Cancer registry and death certificates	Height and weight were measured	BMI	Incidence, colorectal cancer, men	Age, nutritional factors (nos), residence, smoking status
		39/				Incidence, colorectal cancer, women	
Engeland, 2005 Norway	Norwegian composite cohort consisting of 3 groups, Prospective Cohort, Age: 20-74 years, M/W	24 130/ 1 999 978 23 years	Health survey, cancer registry, death registry	Height and weight measured by trained staff	BMI	Incidence, colorectal cancer, women	Age, birth cohort
		22 987/				Men	
		16 638/				Incidence, colon cancer, women	
		13 805/				Incidence, colon cancer, men	
		9 182/				Incidence, rectum cancer, men	
		7 492/				Incidence, rectum cancer, women	
Rapp, 2005 Austria	VHM&PP, Prospective Cohort, Age: 19-94 years, M/W	271/<br><br>1 450 000 person-years	Local physicians	Recorded by medical staff	BMI	Incidence, colon cancer, women	Age, smoking status, occupational group
		260/				Incidence, colon cancer, men	
		138/				Incidence, rectal cancer, men	
		133/				Incidence,	

						rectal cancer, women	
Otani, 2005<br>COL01891<br>Japan	JPHC study-cohort I and II,<br>Prospective Cohort,<br>Age: 40-69 years,<br>M/W	626/<br>102 949<br>9 years	Population registries		Height	Incidence, colorectal cancer, men	Age, alcohol consumption, smoking status, miso soup intake, public health centre area, refraining from salty foods and animal fats
		360/				Incidence, colorectal cancer, women	
Lin, 2004<br>USA	WHS,<br>Prospective Cohort,<br>Age: 45- years,<br>W,<br>professionals	202/<br>36 876<br>8.7 years			BMI	Incidence, colorectal cancer, women	Age, alcohol consumption, family history of specific cancer, history of previous polyp and prior endoscopy, menopausal status, physical activity, randomized treatment assignment, red meat intake, smoking status, aspirin use, postmenopausal hormone use, total energy
		158/				Incidence, colon cancer	
		83/				Incidence, colon, proximal	
		75/				Incidence, colon, distal	
Moore, 2004<br>USA	FHS,<br>Prospective Cohort,<br>Age: 30-79 years,<br>M/W,<br>members of original Framingham study	306/	Self-report, health check, National Death Index	Height and weight were measured	BMI<br><br>Waist-circumference	Incidence, colon cancer	Age, sex, alcohol consumption, educational level, physical activity, smoking habits, height
		97/				Incidence, proximal colon cancer, age: 30-54 yrs	
		91/				Age: 55-79 yr	
		56/				Incidence, distal colon cancer, age: 30-54 yrs	
		53/				Age: 55-79 yr	
Sanjoaquin, 2004<br>UK	OVS,<br>Prospective Cohort,<br>Age: 18-89 years,<br>M/W	92/<br>10 998<br>17 years	Population/invitatio n	Self-reported	BMI	Incidence, colorectal cancer,	Age, sex
							Alcohol consumption, smoking habits
Wei, 2004<br>USA	NHS,<br>Prospective Cohort,<br>W,<br>nurses	672/<br>87 733<br>24 years	Self-report, medical records and National Death Index	Self-reported	BMI	Incidence, colon cancer,	Age, family history, BMI, physical activity, beef, pork or lamb as a main dish, processed meat, alcohol,
		204/				Incidence,	

						rectal cancer	calcium, folate, height, pack-years smoking before age 30, history of endoscopy
	HPFS,<br>Prospective Cohort,<br>M,<br>Health professionals	135/<br>46 632<br>14 years				Incidence,<br>rectal cancer	
		467/				Incidence,<br>colon cancer	
	HPFS & NHS	1123/<br>134 356				Incidence,<br>colon cancer,	
		336/				Incidence,<br>rectal cancer	
		1459/				Incidence,<br>colorectal cancer	
MacInnis, 2004<br>Australia	MCCS, Prospective Cohort,<br>Age: 27-75 years,<br>M	153/<br>16 566<br><br>145 433<br>Person years	Cancer registry		Height	Incidence,<br>colon cancer	Age, sex, country of birth,
Saydah, 2003<br>USA	CLUE II,<br>Nested Case Control,<br>Age: 45- years,<br>M/W	173/<br>346 controls	Cancer registry	Self-report	BMI	Incidence,<br>colorectal cancer,	Age, sex, date of blood draw, race, time since last meal
		132/				Incidence,<br>colon cancer,	
		126/				Incidence,<br>rectal cancer	
Shimizu, 2003<br>Japan	TCCJ,<br>Prospective Cohort,<br>Age: 35- years,<br>M/W	108/<br>29 051<br>8 years	Hospital records and cancer registry	Self-reported	Height	Incidence,<br>colon cancer,<br>men	Age, alcohol consumption, educational level, physical activity, smoking habits, height
		93/				Incidence,<br>colon cancer,<br>women	
		59/				Incidence,<br>rectal cancer,<br>men	

		41/				Incidence, rectal cancer, women	
Gunnell, 2003 UK	Caerphily study, Prospective cohort study, Age:45-59 years	38/ 2512 21 years	Central personal register	Measured	Height	Incidence, colorectal cancer	Age, smoking status, BMI, occupation, household size,unemployment
Terry, 2002 Canada	CNBSS, Prospective Cohort, Age: 40-59 years, W	527/ 89 835 936 433 person-years	National Mortality Database and to the Canadian Cancer Database	Self-reported	BMI	Incidence, colorectal cancer	Age, educational level, oral contraceptive use, parity, physical activity, smoking habits, HRT
		363/				Incidence, colon cancer	
		172/				Incidence, proximal colon cancer	
		164/				Incidence, rectal cancer	
		148/				Incidence, distal colon cancer	
Terry, 2001 Sweden	SWSC, Prospective Cohort, Age: 40-76 years, W	460/ 61 463 588 270 person-years	Cancer registry	Self-reported	BMI	Incidence, colorectal cancer,	Age, alcohol consumption, educational level, energy intake, red meat intake, vitamin d, calcium, folate, total fat, vitamin c
		291/				Incidence, colon cancer,	
		159/				Incidence, rectal cancer,	
		118/				Incidence, proximal colon cancer,	
		101/				Incidence, distal colon cancer,	
Kaaks, 2000 USA	NYUWHS, Nested Case Control,	100/ 196 controls	Active follow up by questionnaire; cancer registry and	Self-reported height and weight	BMI	Incidence, colorectal cancer	Age, menopausal status, reproductive factors, smoking status, time

	Age: 35-65 years,<br>W,<br>Screening Program		national death<br>Index				
		73/<br>144<br>controls				Incidence,<br>colon cancer	
Folsom, 2000<br>USA	IWHS,<br>Prospective Cohort,<br>Age: 55-69 years,<br>W	462/<br>31 702<br>10 years	SEER	Self-reported	Waist-circumference<br><br>Waist to hip ratio	Incidence,<br>colon cancer	Age, BMI, age at first child birth, alcohol consumption, estrogen use, educational level, smoking status, physical activity, pack-years of cigarette, energy intake, intakes of fish, fruit, red meat, vegetables whole grain, vitamin use
Ford, 1999<br>USA	NHANES I,<br>Prospective Cohort,<br>Age: 25-74 years,<br>M/W	222/<br>13 420<br>19 years	Multistage stratified sampling design	Weight and height were measured	BMI	Incidence,<br>colon cancer	Age
						Incidence,<br>colon cancer,<br>men	
						Incidence,<br>colon cancer,<br>women	
Schoen, 1999<br>USA	CHS,<br>Prospective Cohort,<br>Age: 65- years,<br>M/W	102/<br>5 849	Medicare enrolment lists	Weight and height were measured	BMI	Incidence,<br>colorectal<br>cancer	Age, sex, physical activity
Singh, 1998<br>USA	AHS,<br>Prospective Cohort,<br>Age: 25- years,<br>M/W,<br>Seventh-day Adventists	142/<br>32 051<br>178 544<br>person-years	Hospital records and cancer registry	Self-reported	BMI	Incidence,<br>colon cancer	Age, sex, family history of specific cancer
		83/				Incidence,<br>colon cancer,<br>women	
		59/				Incidence,<br>colon cancer,<br>men	
Albanes, 1988<br>USA	NHANES I<br>Age: 25-74 years,<br>M/W	62/<br>12 554<br>10 years	Hospital records, death certificate	Measured	Height	Incidence,<br>colorectal<br>cancer, men	Age

		67/				Incidence, colorectal cancer, women	
Tulinius, 1997 Iceland	ICRF, Prospective Cohort, M/W	193/ 22 946	Population registry	Anthropometrics measured	BMI<br><br>Weight	Incidence, colorectal cancer, men	Age
Kato, 1997 USA	NYUWHS, Prospective Cohort, Age: 34-65 years, W	100/ 14 272 105 044 person-years	Questionnaire, medical records, cancer registries		Height	Incidence, colorectal cancer	Age, place at enrolment
Hebert, 1997 USA	PHS Age: 40-84 years M	341/ 22 071 12 years	Medical records	Self-reported	Height	Incidence, colorectal cancer	Age, beta-carotene, BMI, Aspirin use, smoking, alcohol consumption, physical activity
						Incidence, colon cancer	
Martínez, 1997 USA	NHS, Prospective Cohort, Age: 30-55 years, W, Registered nurses	161/ 89 448 1 012 375 person-years	Cancer registry	Self-reported	Waist to hip ratio	Incidence, colon cancer, 1986-1992 follow-up	Age, alcohol consumption, aspirin use, BMI, cigarette smoking, family history of specific cancer, postmenopausal hormone use, red meat intake
Chyou, 1996 USA	HHP, Prospective Cohort, M, Japanese ancestry	330/ 8 006 19 years	Hospital records + cancer registry	Self-reported	BMI	Incidence, colon cancer	Age
		123/				Incidence, rectal cancer	
Thune, 1996 Norway	NHSCD, Prospective Cohort, Age: 20-69 years, M/W	230/ 81 516 1 305 607 person-years	Cancer registry	Height and weight were measured	BMI	Incidence, colon cancer, men	Age
		169/				Incidence, rectal cancer, men	
		99/				Incidence, colon cancer, women	
		55/				Incidence, rectal cancer,	

						women	
Giovannucci, 1995<br>USA	HPFS,<br>Prospective Cohort,<br>Age: 40-75 years,<br>M	117/<br>31 055<br>6 years	Medical records, national Death Index	Questionnaire	Waist-circumference<br><br>Waist to hip ratio	Incidence, colon cancer	Age, alcohol consumption, aspirin use, dietary fibre intake, energy intake, family history of specific cancer, folate intake, history endoscopic screening, methionine intake, physical activity, previous polyp diagnosis, red meat intake, smoking habits
Bostick, 1994<br>USA	IWHS,<br>Prospective Cohort,<br>Age: 55-69 years,<br>W	212/<br>35 216<br>167 447 person-years	SEER		Height	Incidence, colon cancer	Age, energy intake, parity, vitamin a supplement, vitamin E intake
Chyou, 1994<br>USA	HHP,<br>Prospective Cohort,<br>M,<br>Japanese ancestry	289/<br>7 945<br>19 years	Selective service draft registration file		Weight	Incidence, colon cancer	Age
		108/				Incidence, rectal cancer	
Lee, 1992<br>USA	HAHS,<br>Prospective Cohort,<br>Age: 48 years,<br>M	290/<br>17 595<br>26 years	Health examination check	Height and weight were measured	BMI	Incidence, colon cancer,	Age, physical activity, parental history of cancer
Wu, 1987<br>USA	LWC,<br>Prospective Cohort,<br>M/W,<br>Retirement community	68/<br>11 644<br>4.5 years	Population registries	self-administered	BMI	Incidence, colorectal cancer, women	Age
		58/				Incidence, colorectal cancer, men	Alcohol consumption, physical activity, smoking habits

Figure1.

A: Height and colon cancer, per 5 cm

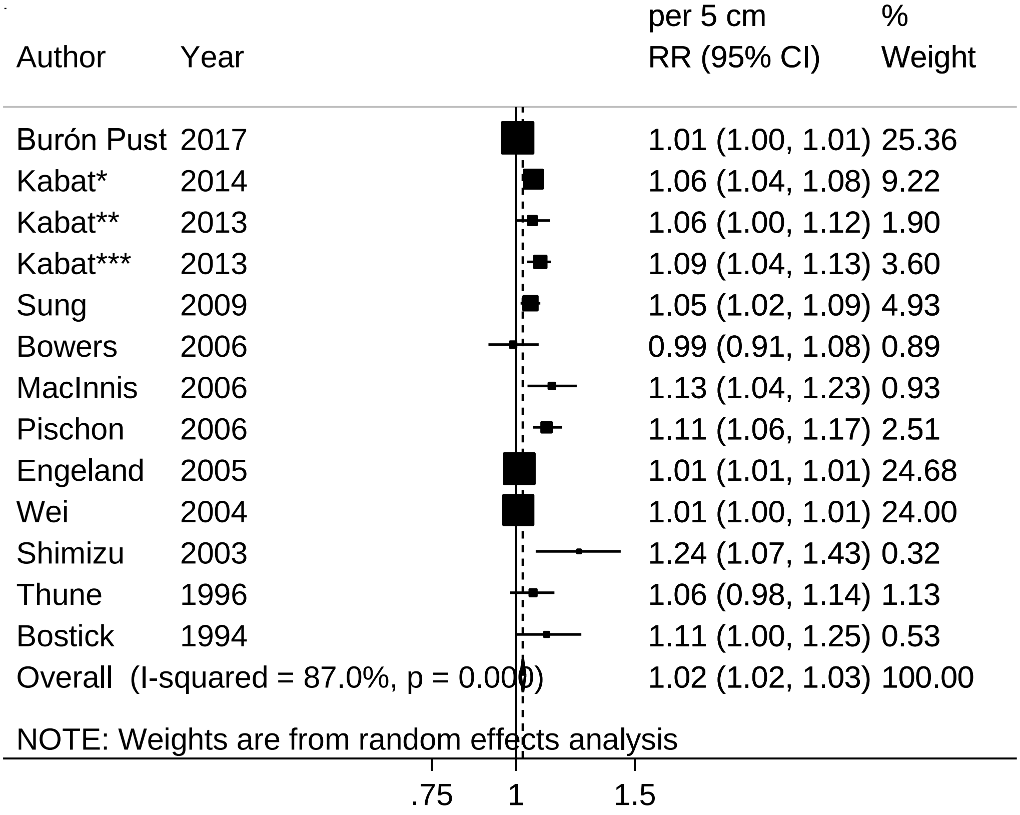

B:Height and colon cancer, nonlinear dose-response

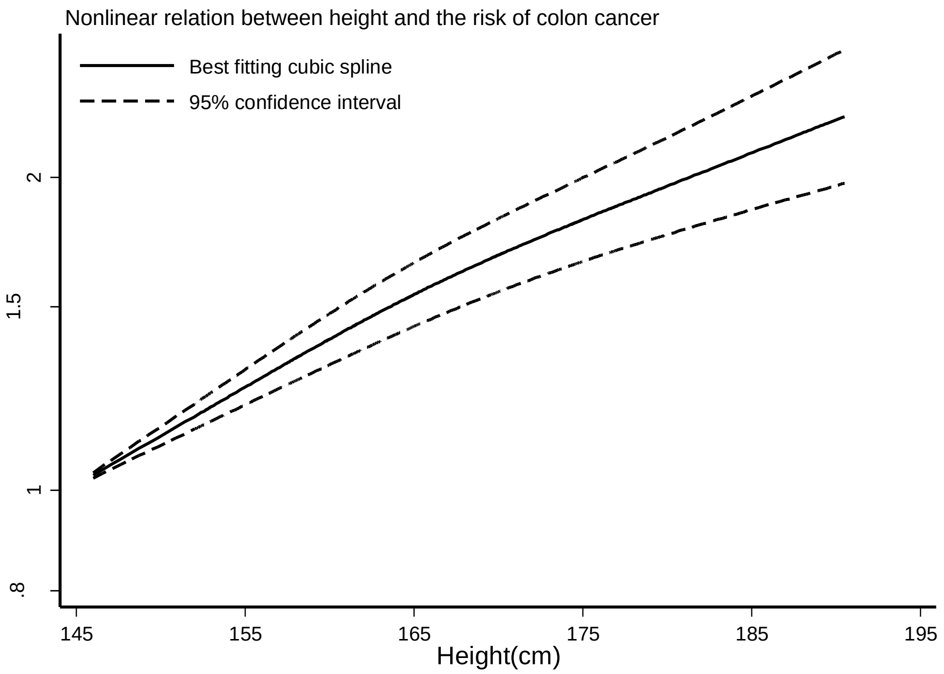

Figure2.

A: Height and proximal colon cancer

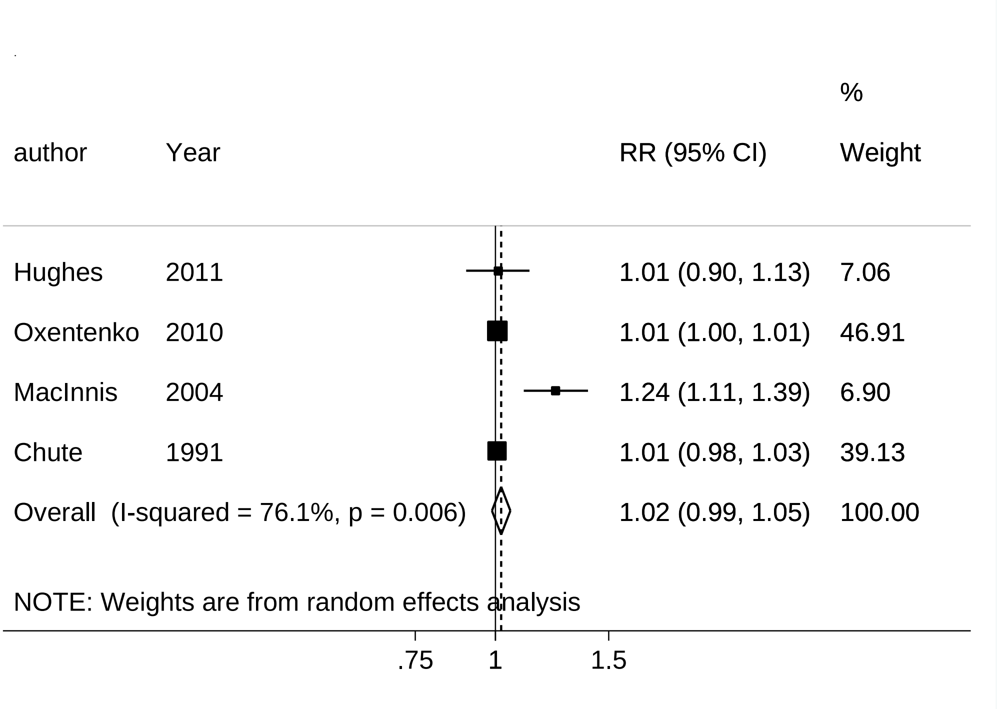

B: Height and proximal colon cancer, nonlinear dose-response

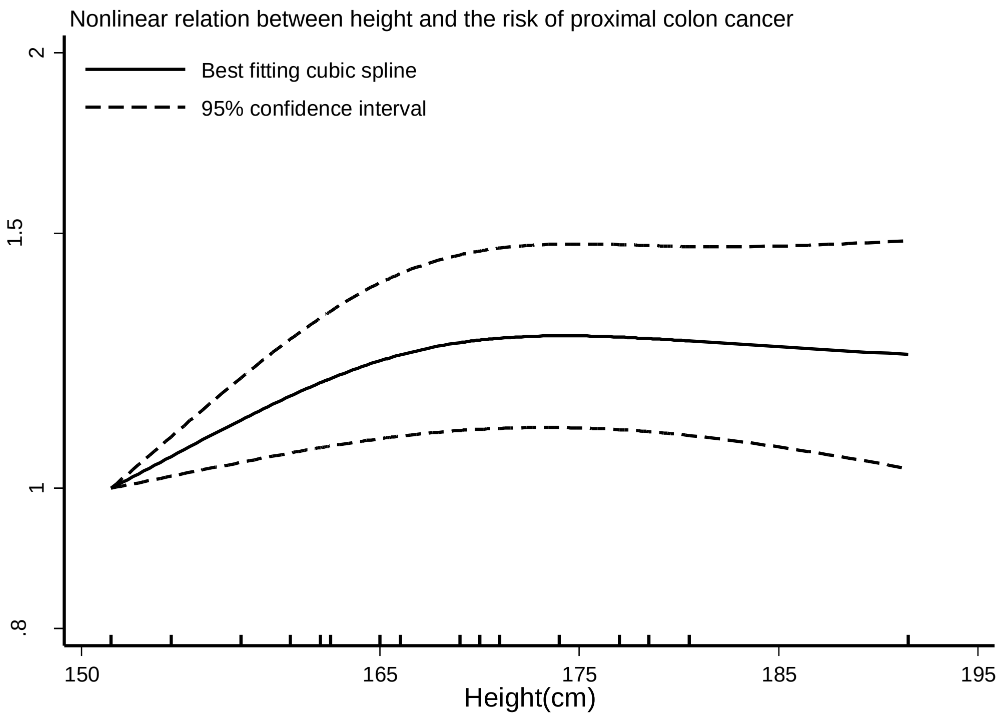

Figure3.

A: Height and distal colon cancer

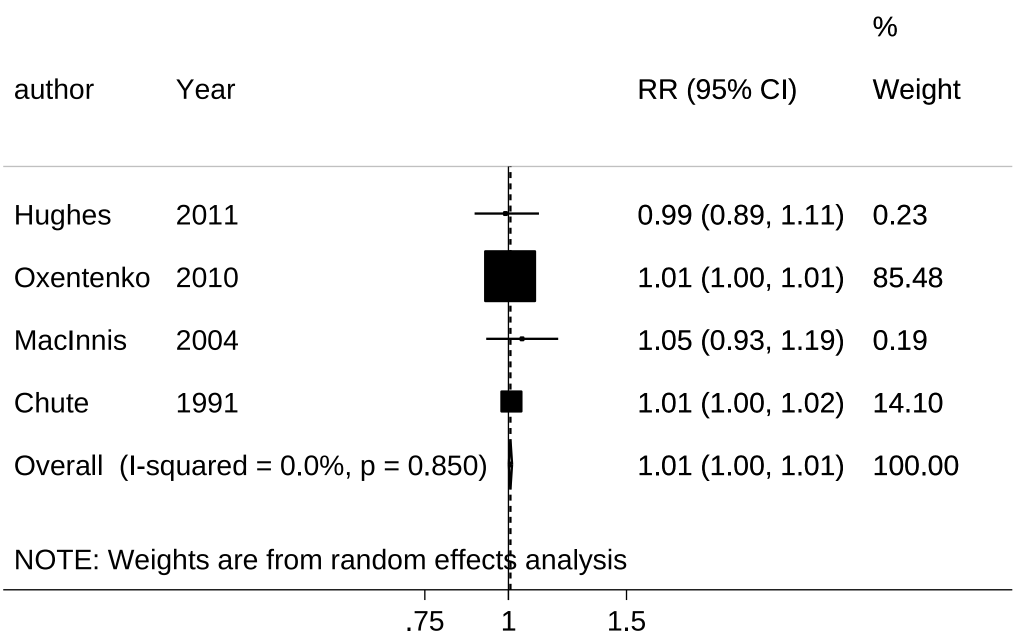

B: Height and distal colon cancer, noninear dose-response

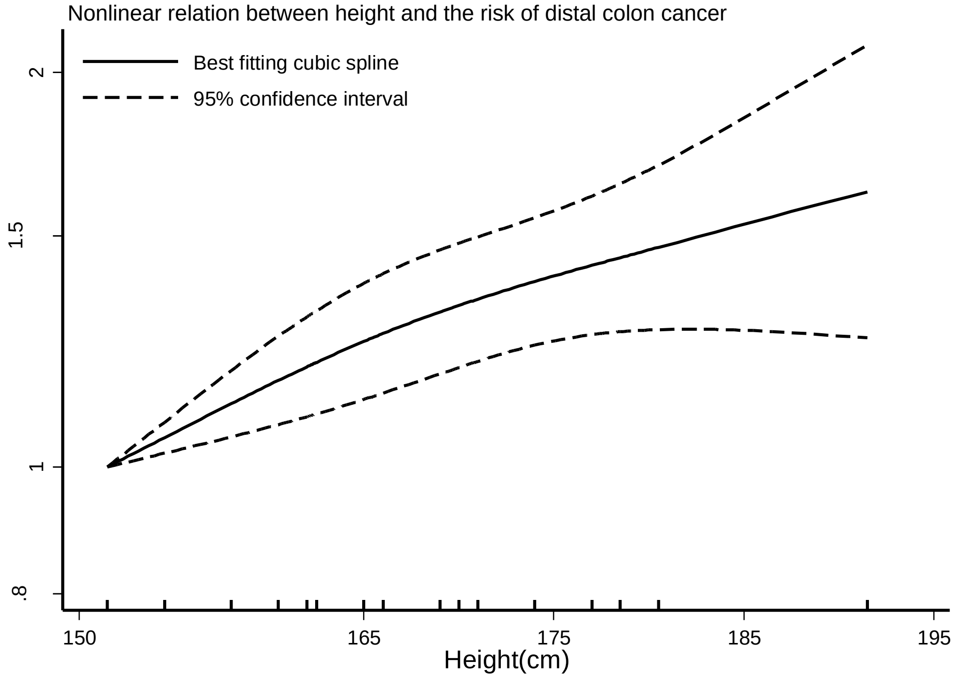

Figure4.

A: height and rectal cancer, per 5 cm

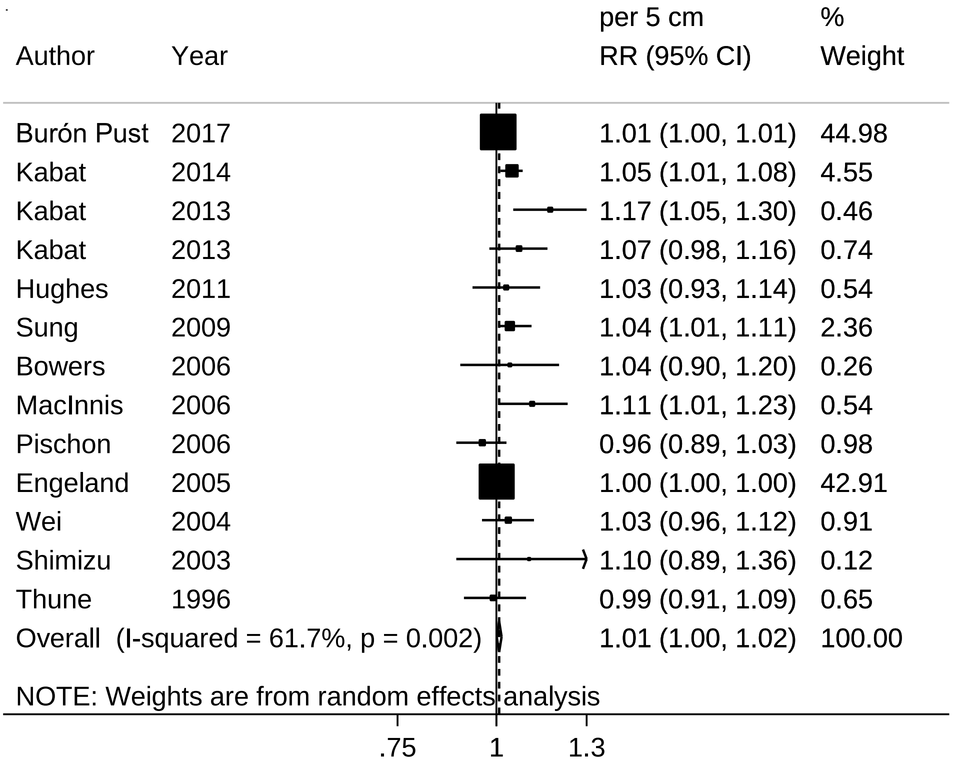

B: height and rectal cancer, nonlinear dose-response

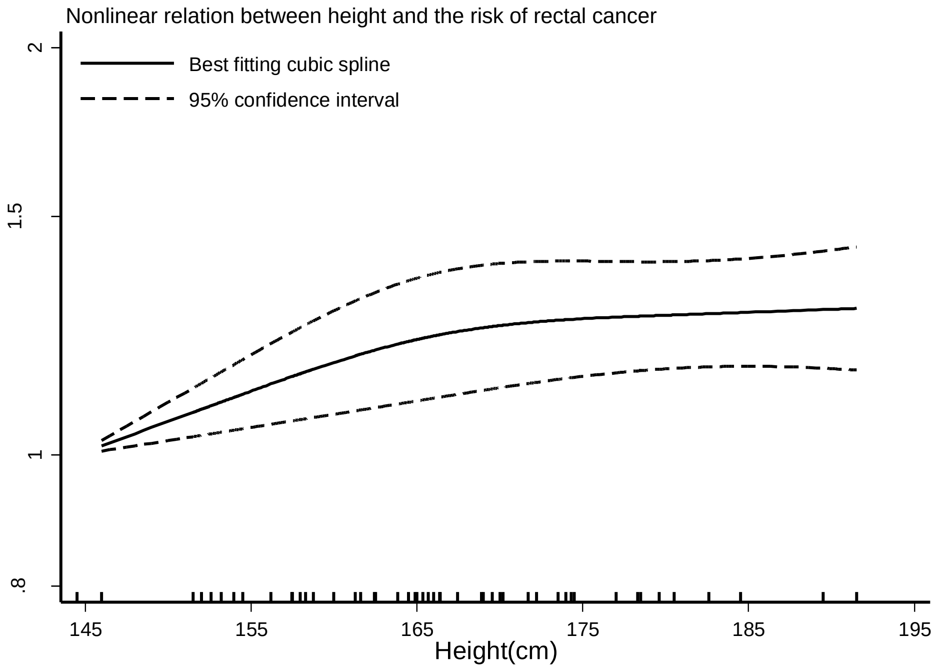

Figure5.

A: Weight and colon cancer

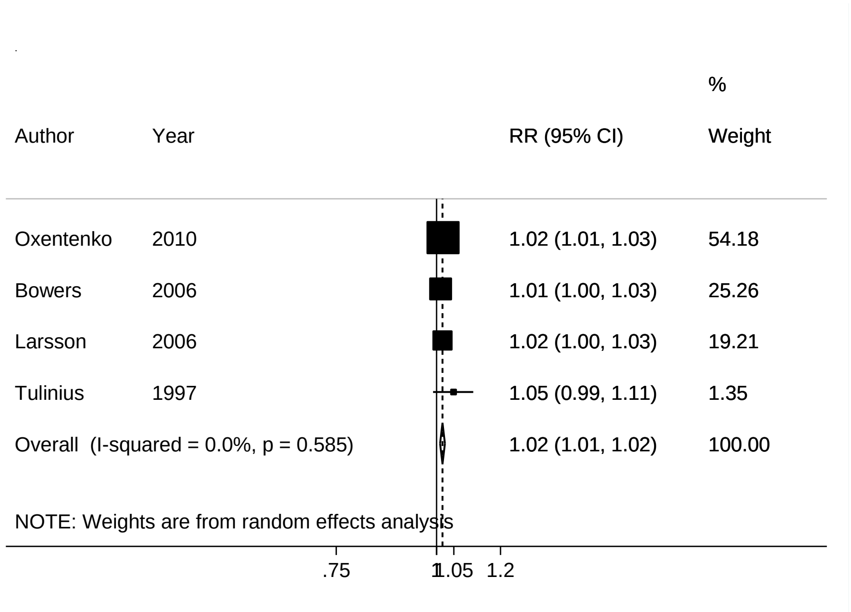

A: Weight and colon cancer, nonlinear dose-response

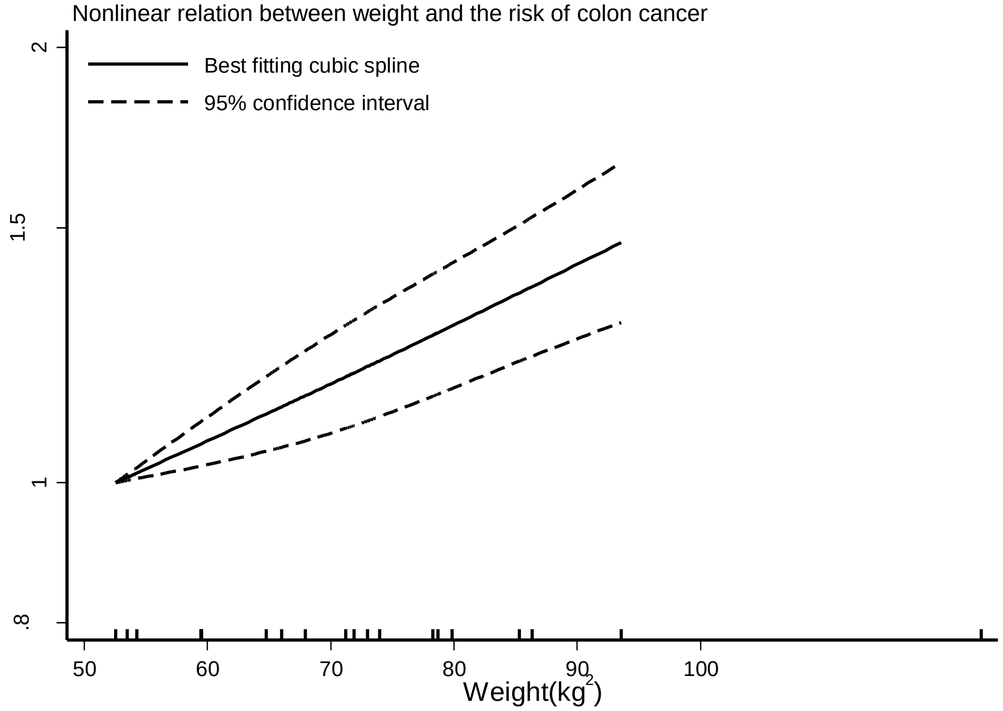

Figure6.

A:weight and proximal colon cancer

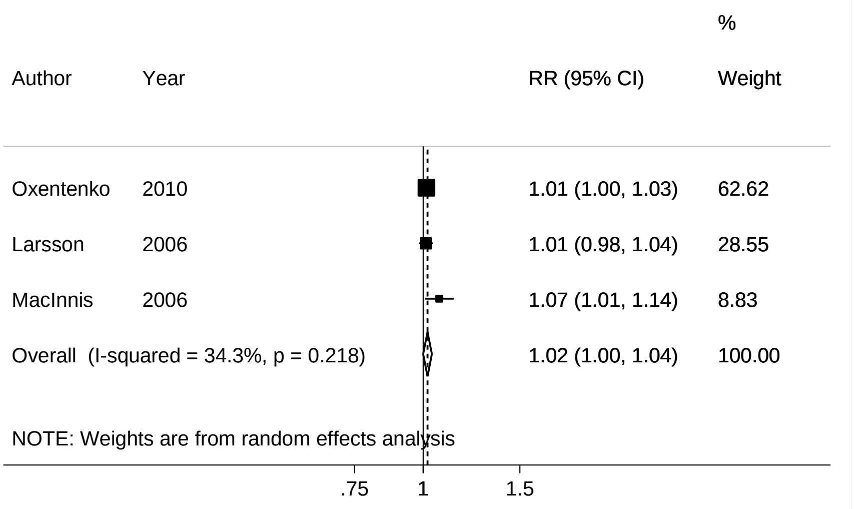

B: weight and proximal colon cancer, nonlinear dose-response

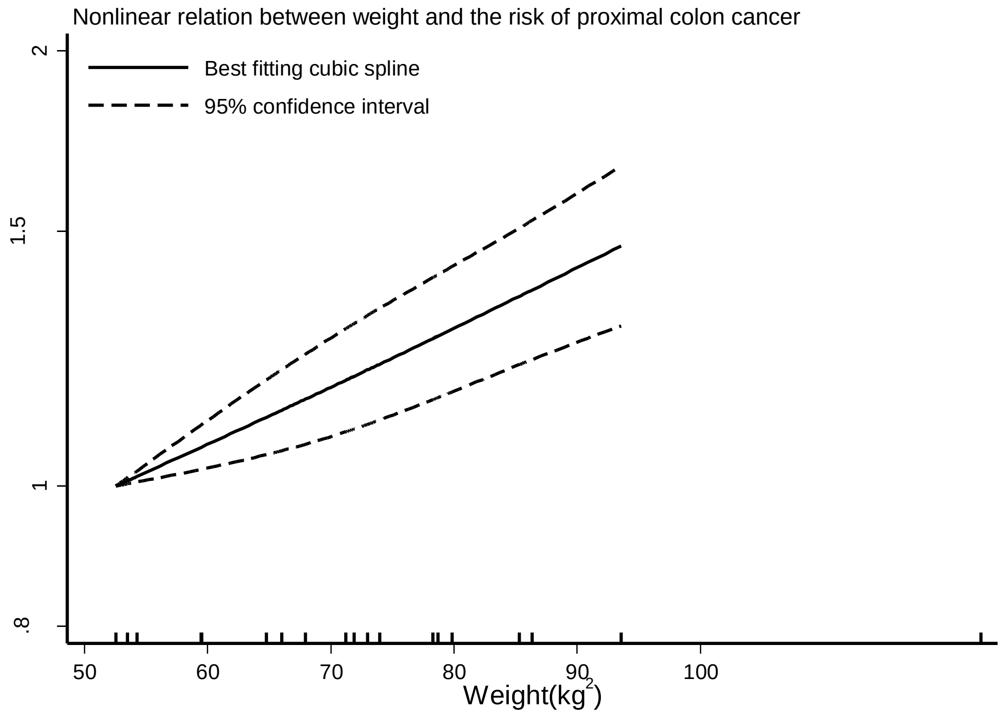

Figure7.

A:weight and distal colon cancer

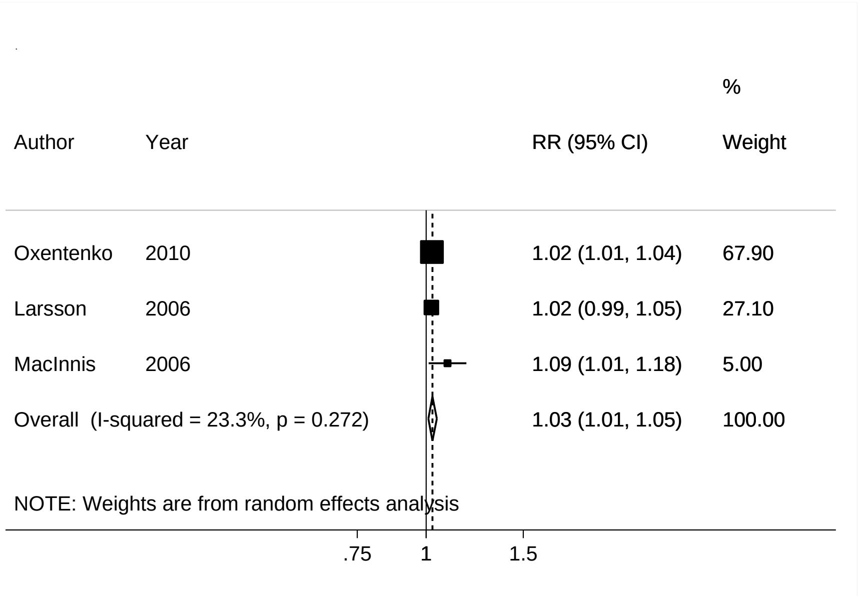

B: weight and distal colon cancer, nonlinear dose-response

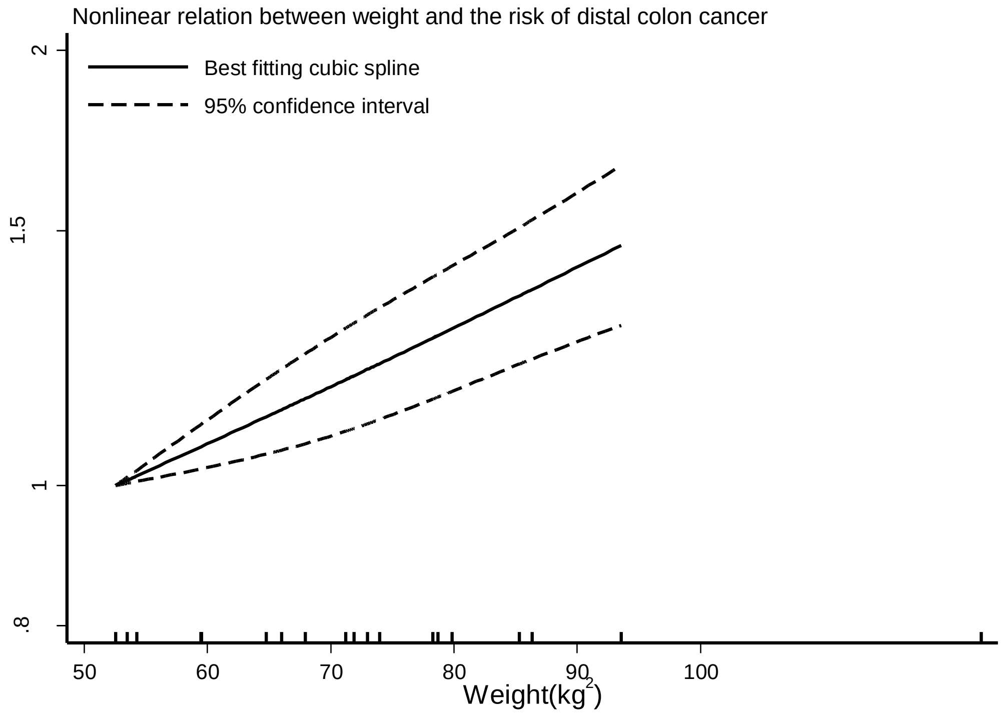

Figure8.

A:weight and rectal cancer

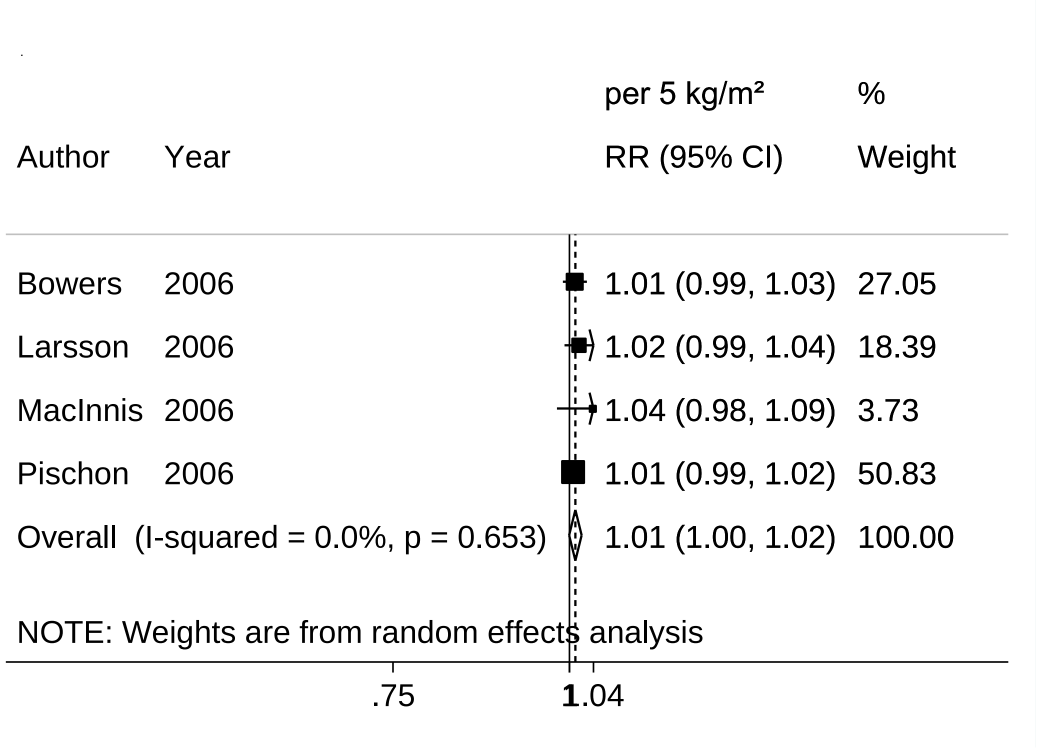

B:weight and rectal cancer, nonlinear dose-response

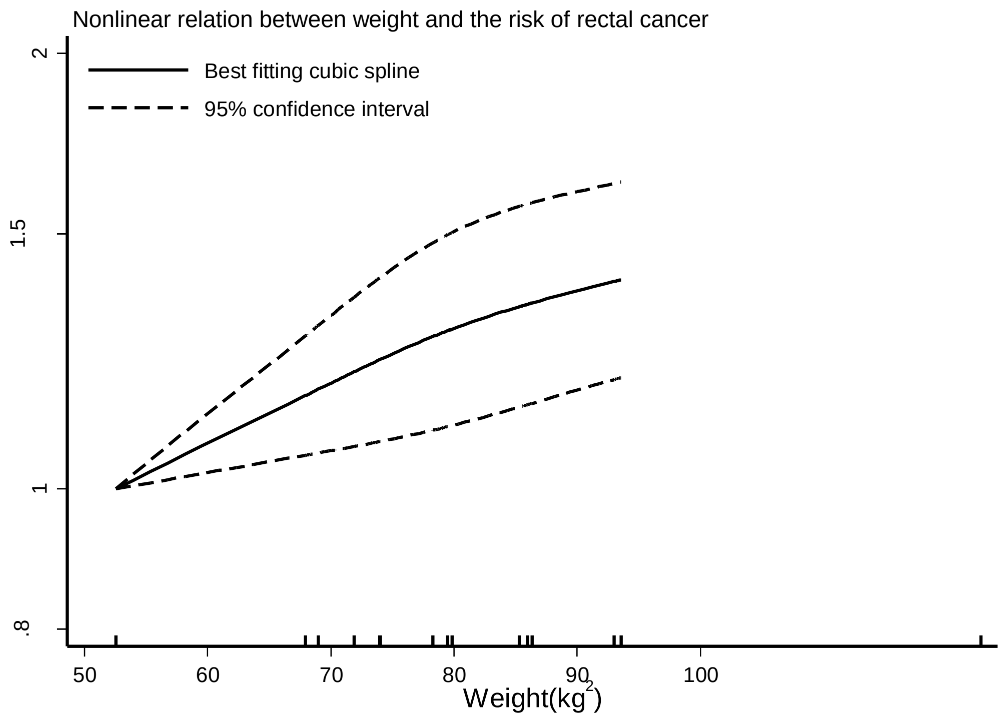
Nonlinear relation between weight and the risk of rectal cancer
Best fitting cubic spline
95% confidence interval
2
1.5
1
.8
50
60
70
80
90
100
Weight(kg2)

Figure9.

A: BMI and colon cancer, per 5 kg/m²

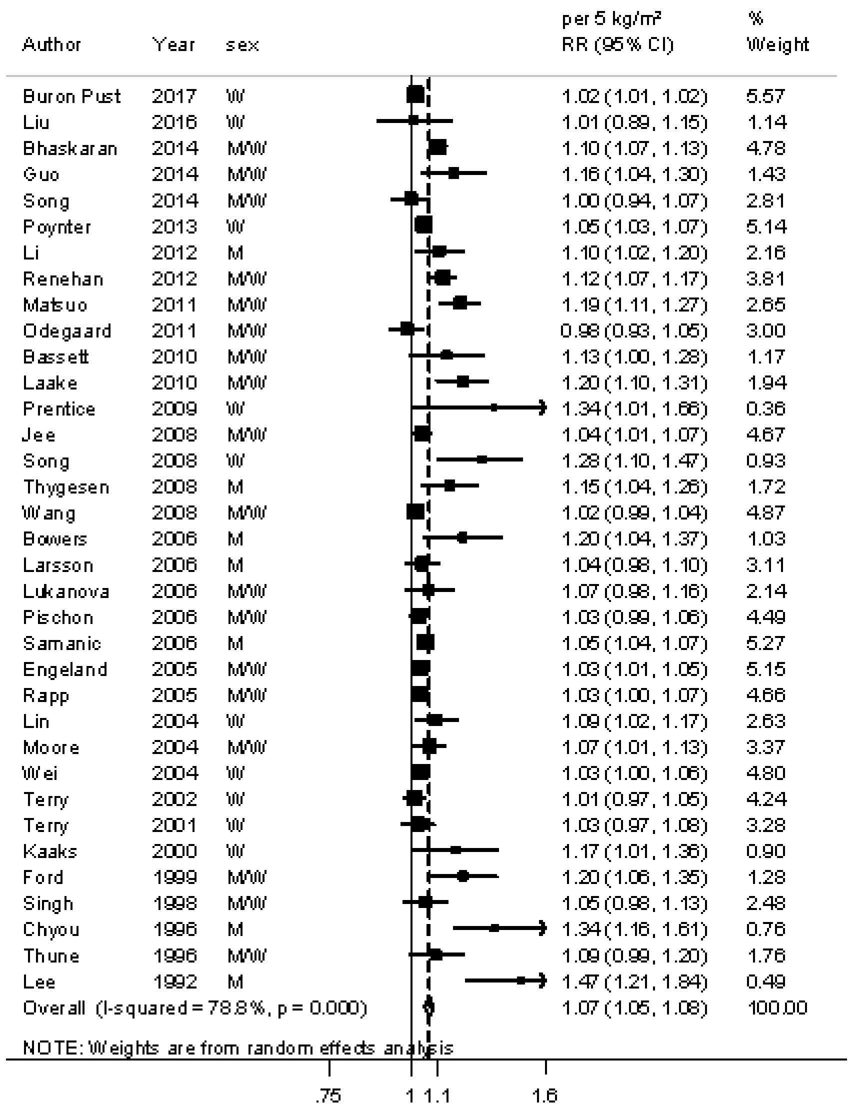

B: BMI and colon cancer, nonlinear dose-response

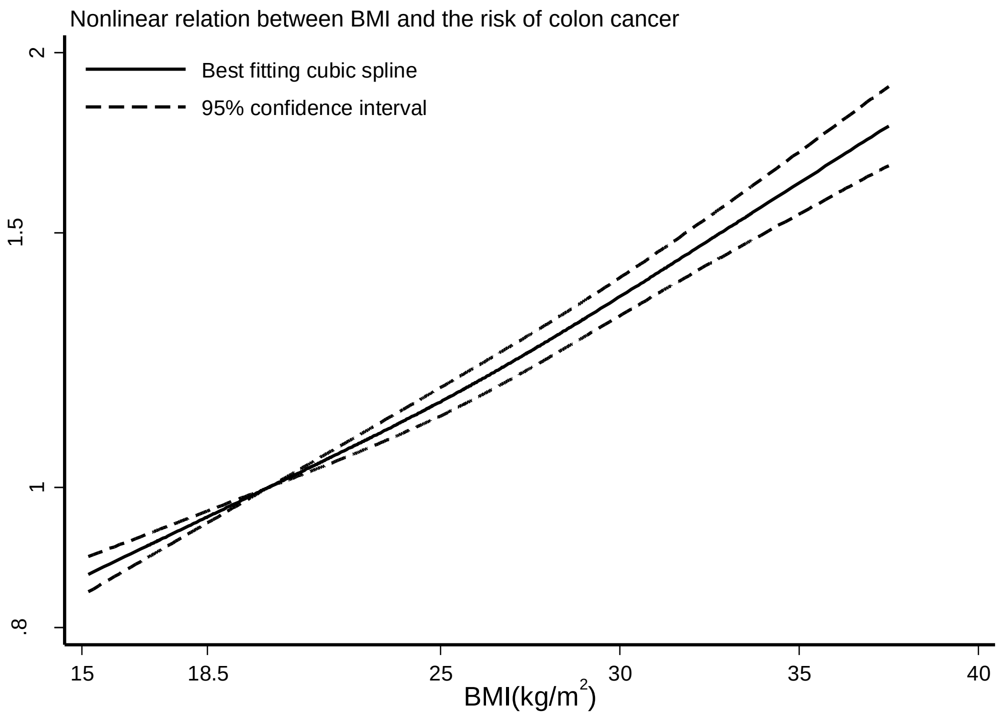

Figure10.

A: BMI and proximal colon cancer

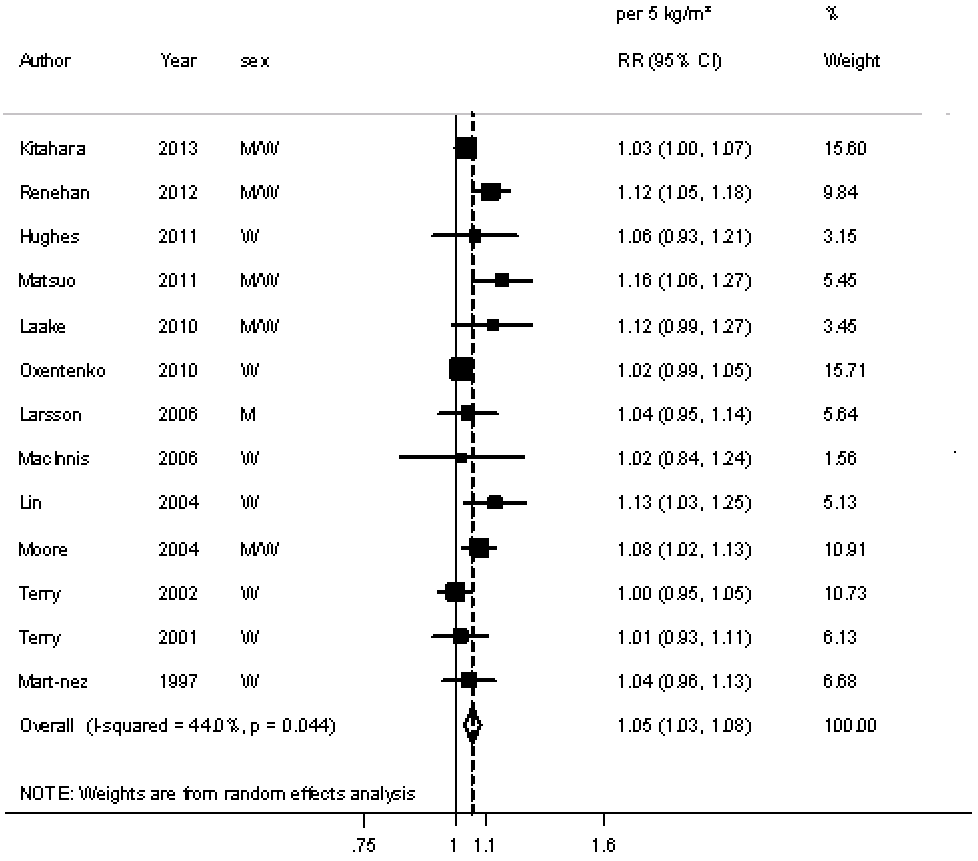

A: BMI and proximal colon cancer, nonlinear dose-response

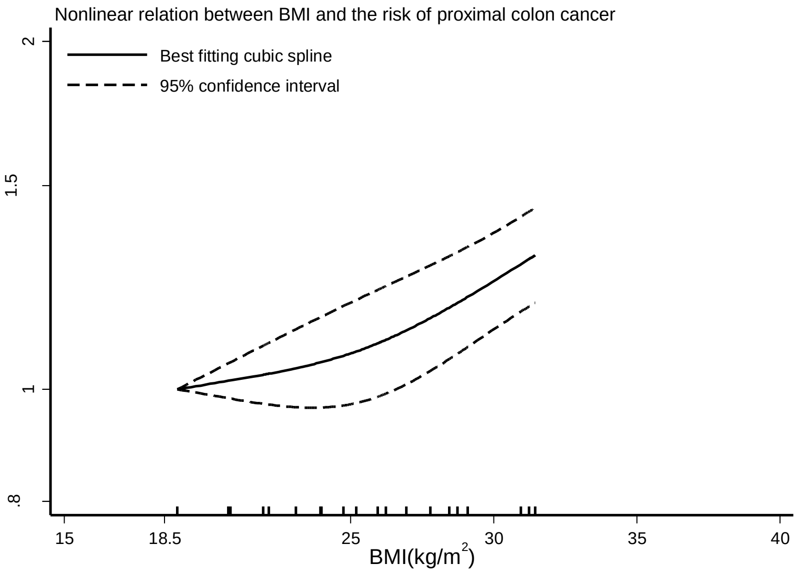

Figure11.

A: BMI and distal colon cancer

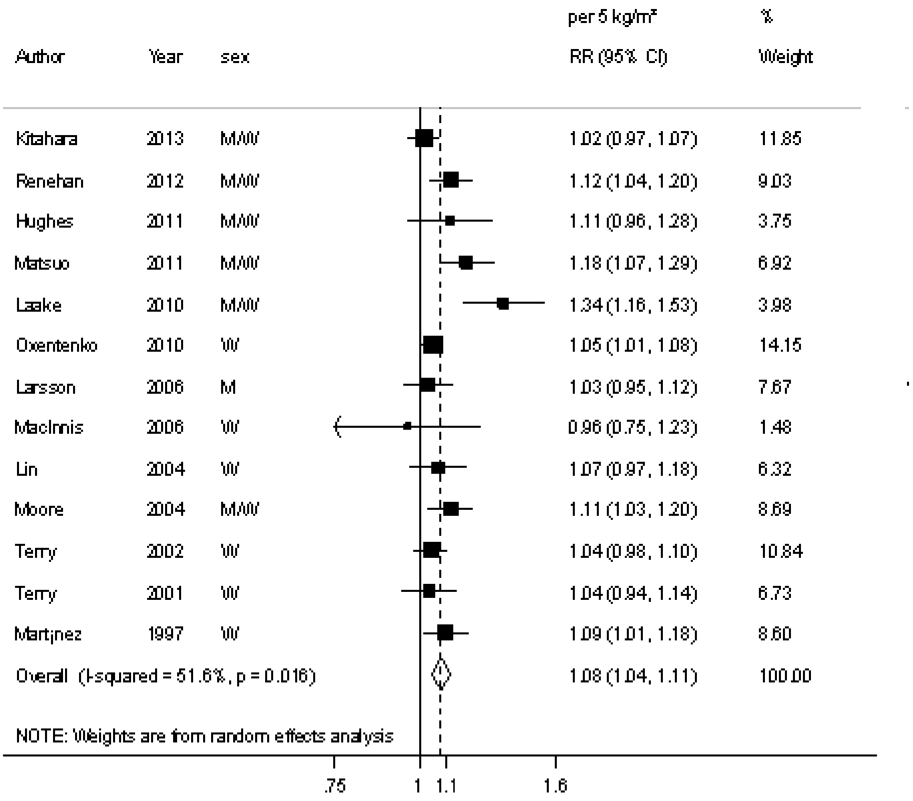

B: BMI and distal colon cancer, nonlinear dose-response

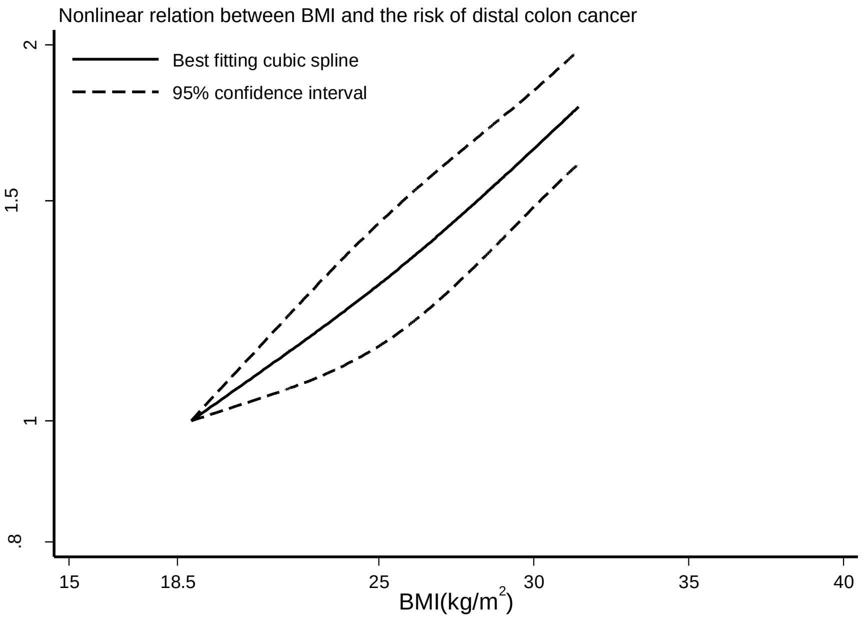

Figure12.

A: BMI and rectal cancer, per 5 kg/m²

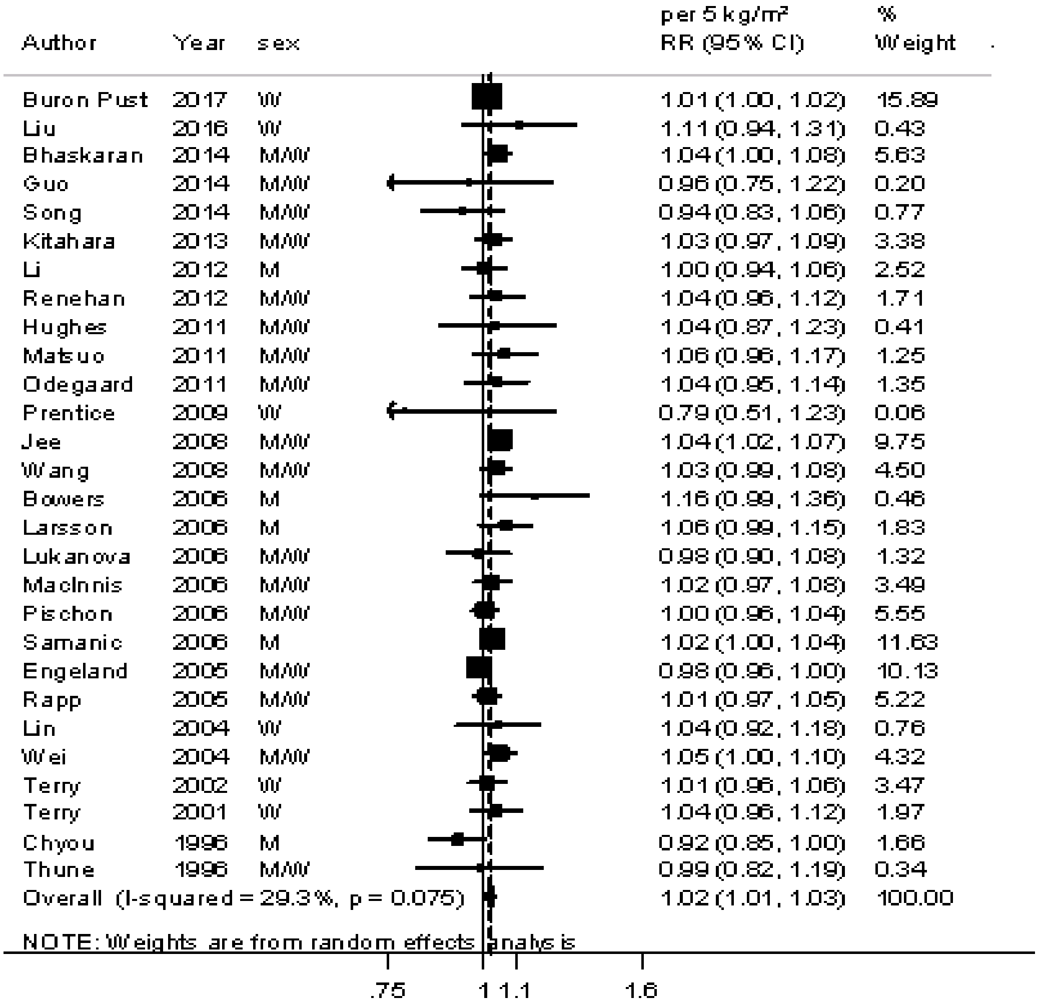

B: BMI and rectal cancer, nonlinear dose-response

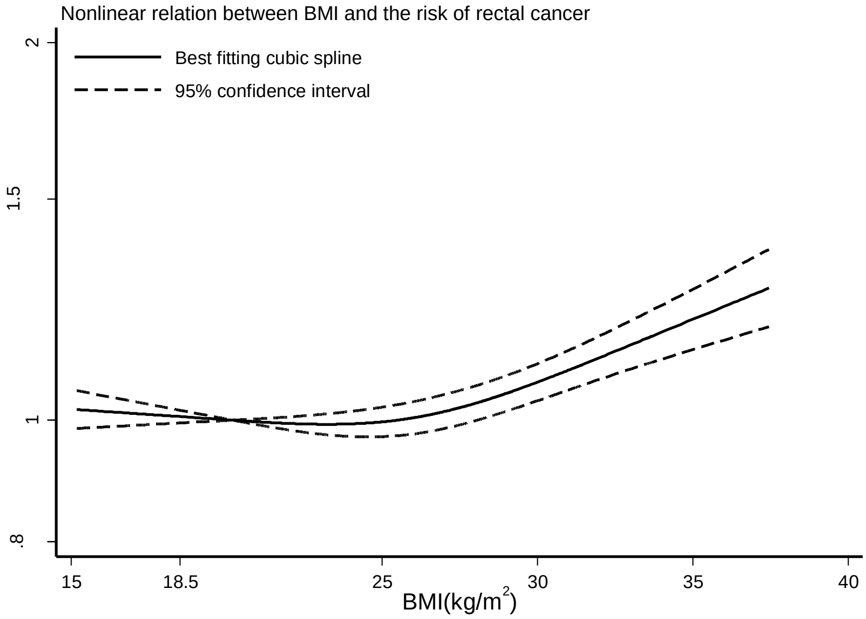

Figure13.

A: Waist circumference and colon cancer, per 10 cm

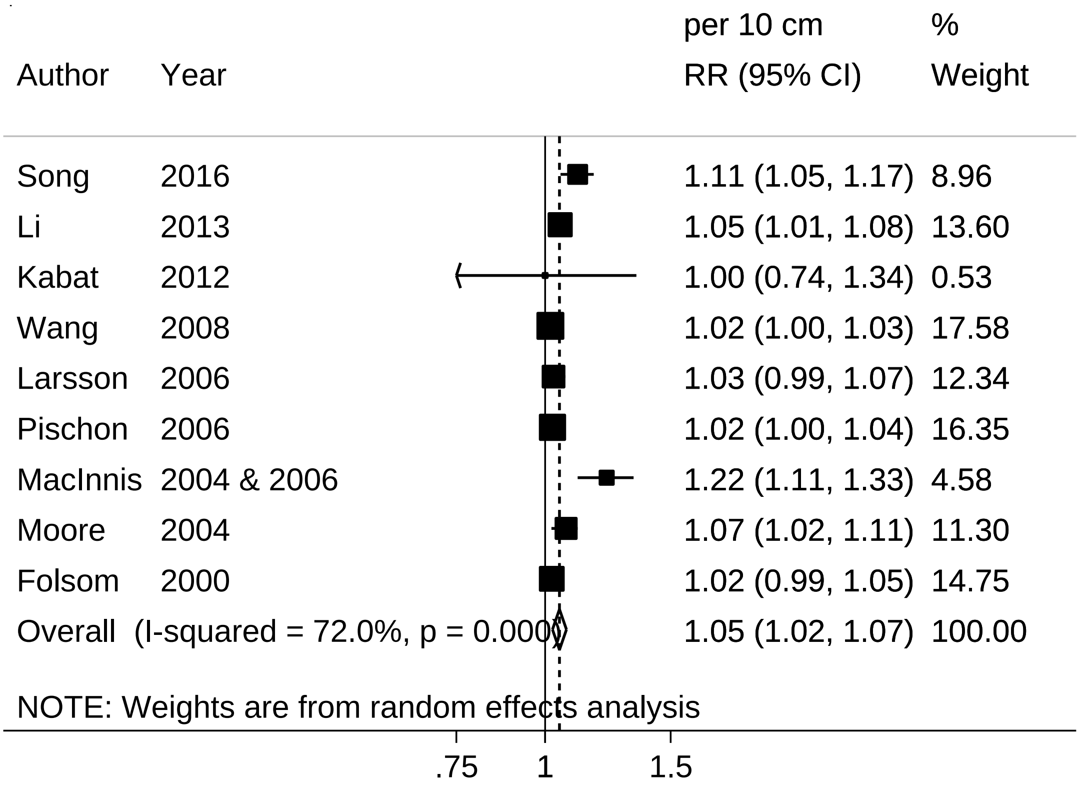

B: waist circumference and colon cancer, nonlinear dose-response

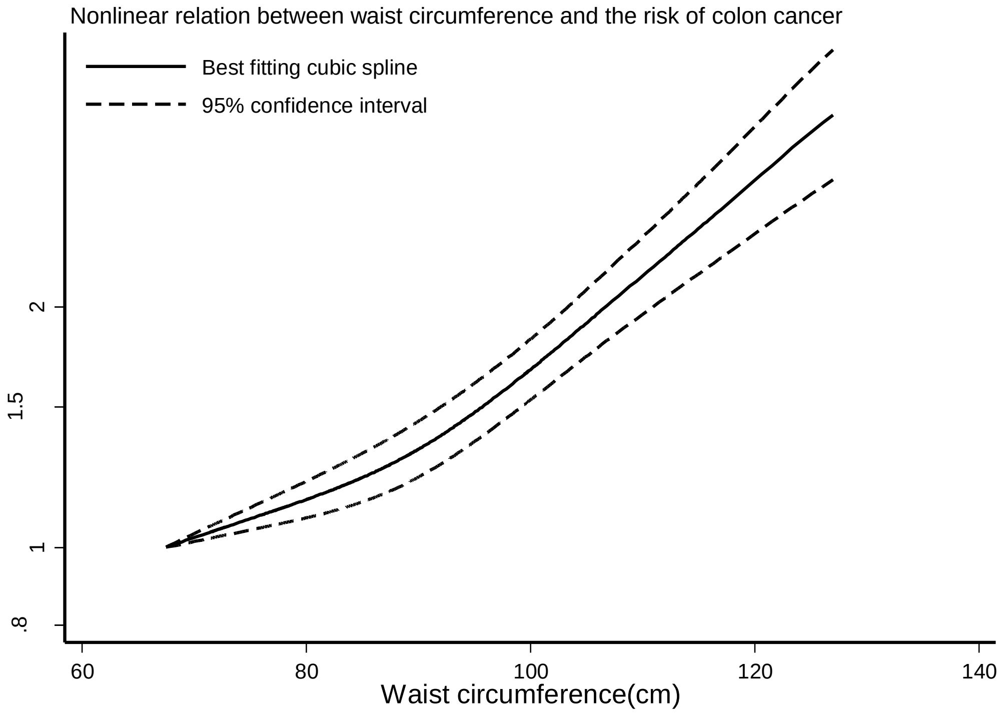

Figure14.

A: waist circumference and proximal colon cancer

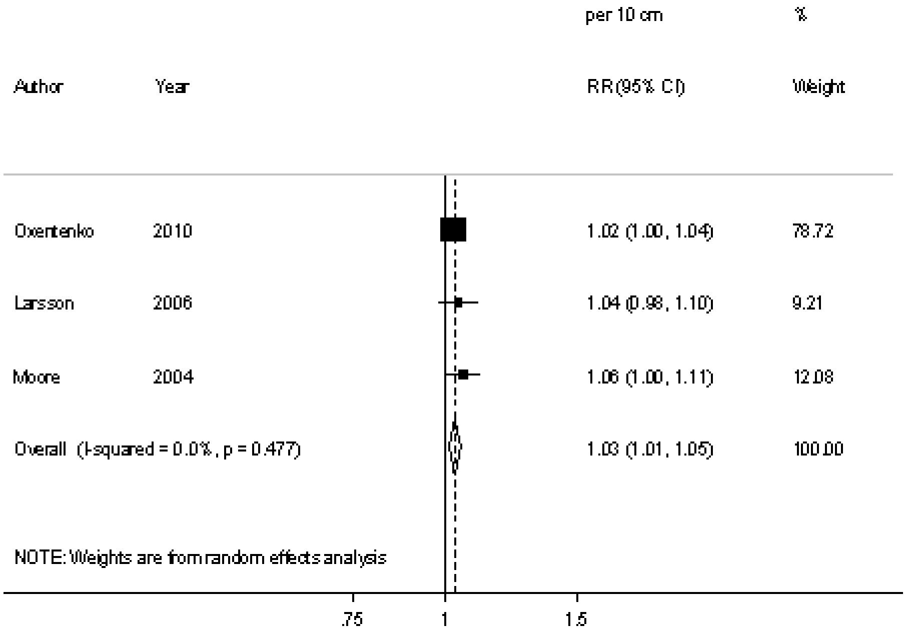

B: waist circumference and proximal colon cancer, nonlinear dose-response

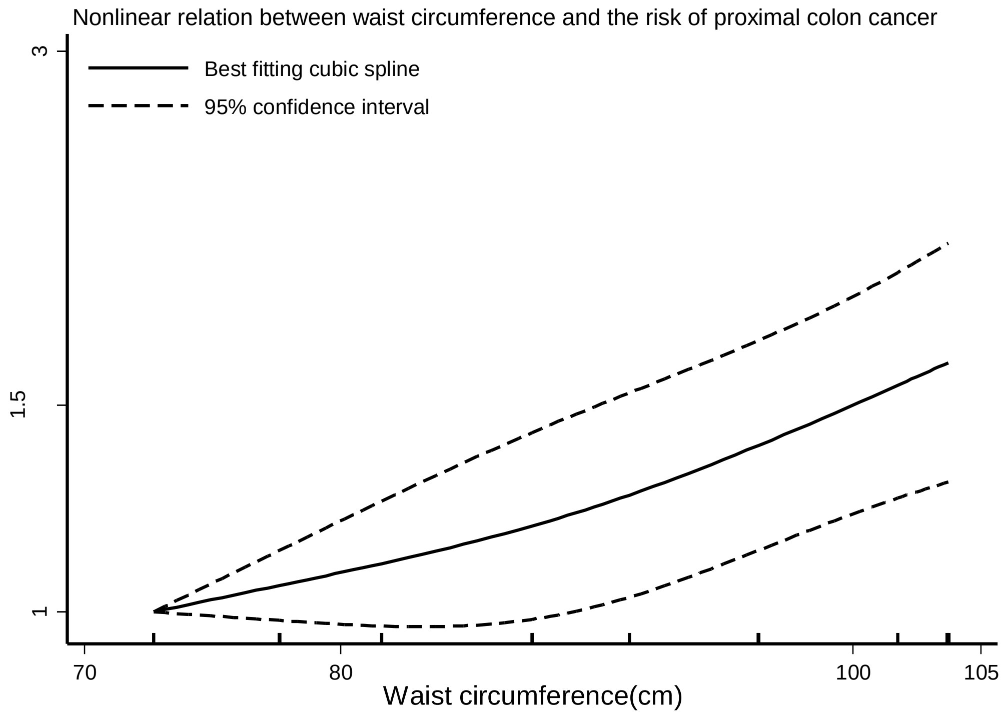

Figure15.

A:waist circumference and distal colon cancer

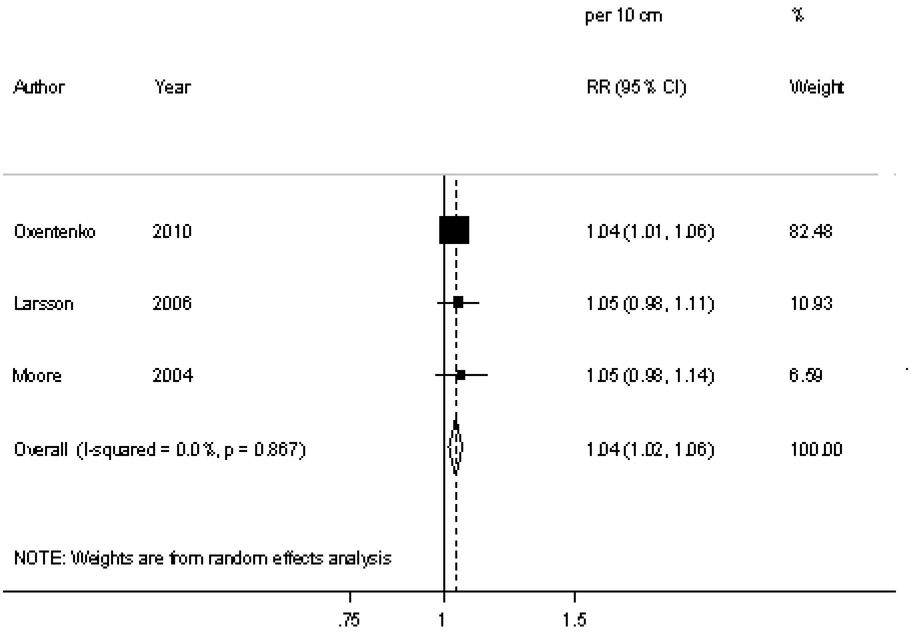

B: waist circumference and distal colon cancer, nonlinear dose-response

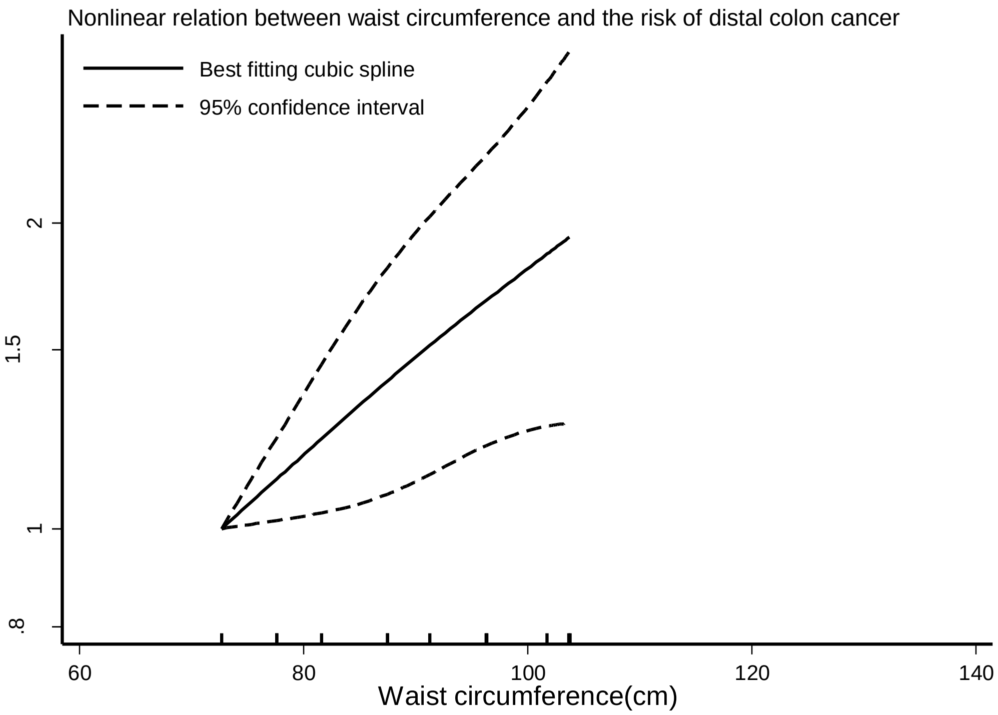

Figure16.

A: waist circumference and rectal cancer, per 10 cm

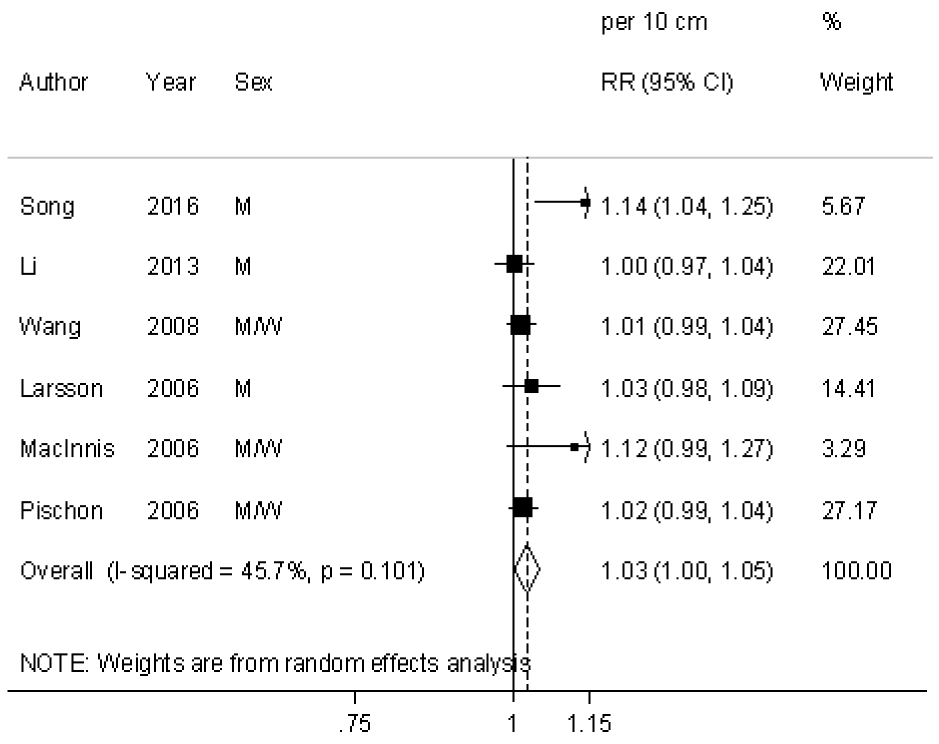

B: Waist circumference and rectal cancer, nonlinear dose-response

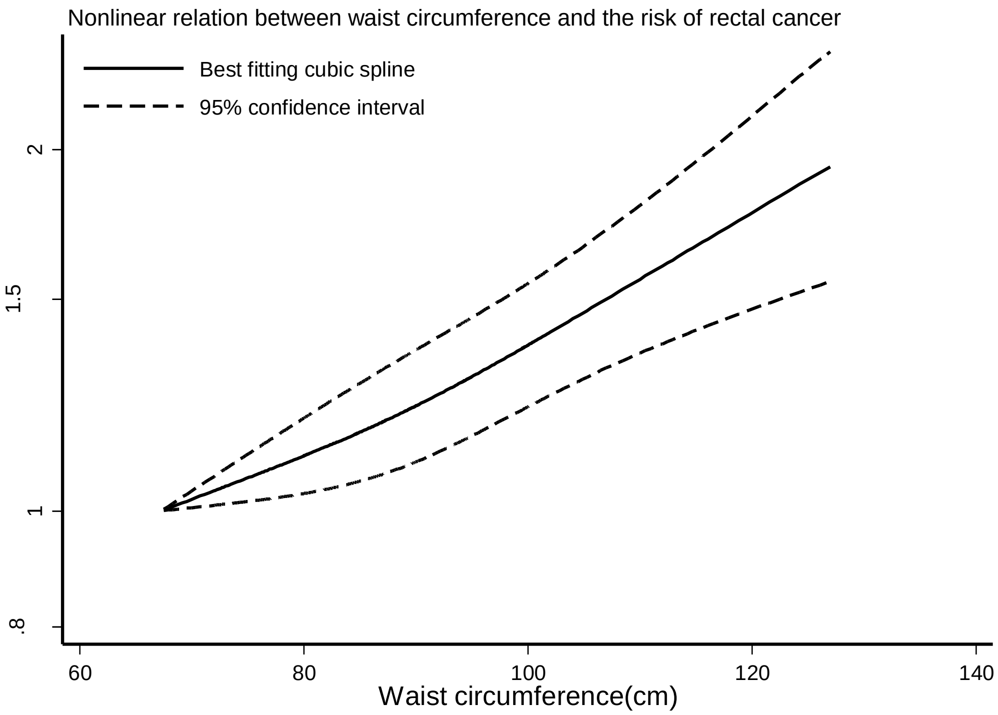

Figure17.

A: waist to hip ratio and colon cancer, per 0.1 unit

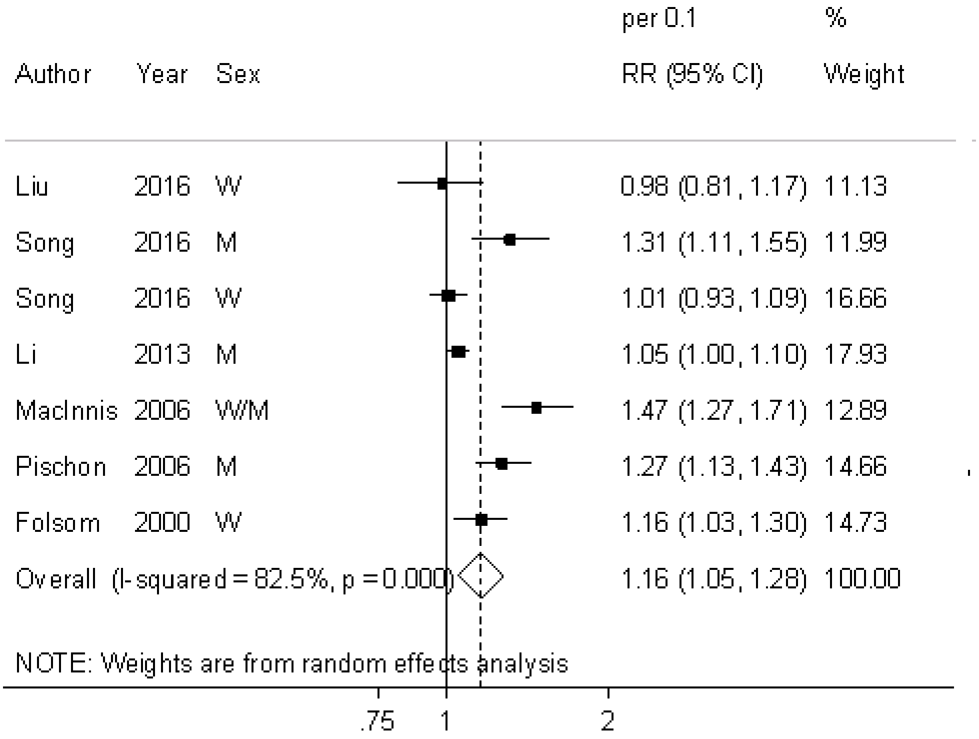

B: waist to hip ration and colon cancer, nonlinear dose-response

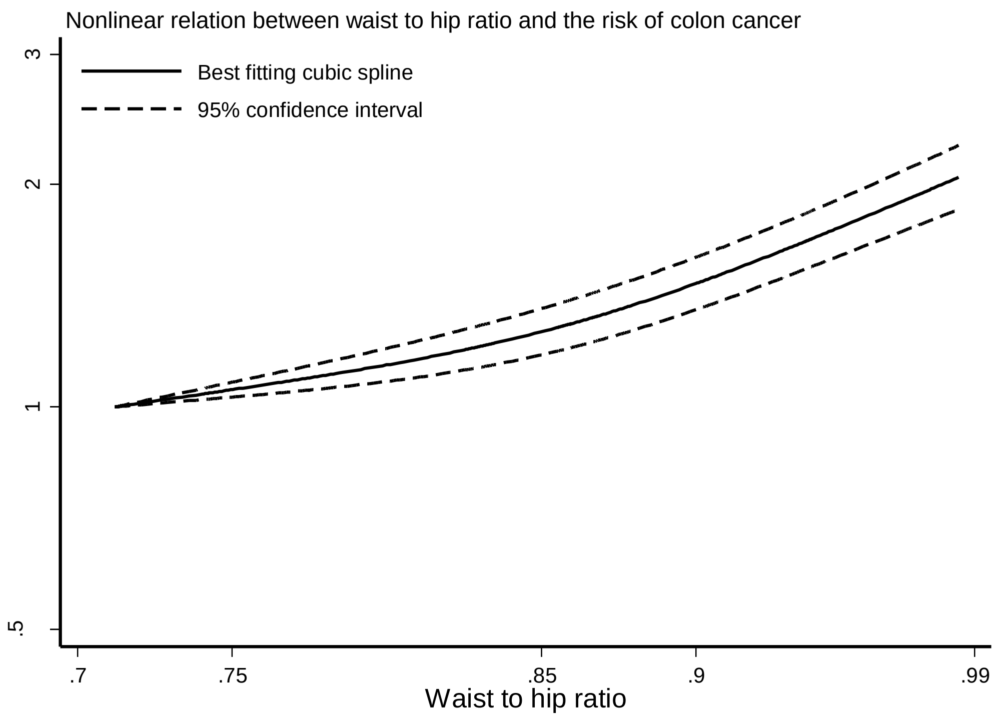

Figure18.

Waist-to-hip ratio and proximal colon cancer

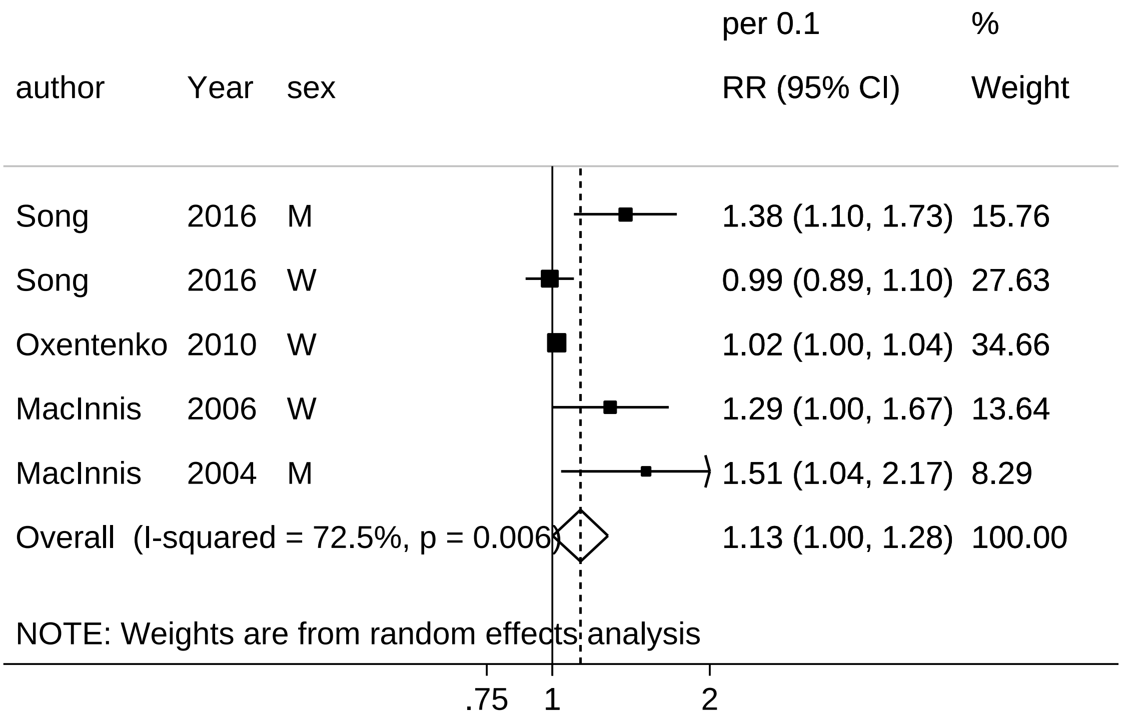

Figure19.

Waist-to-hip ratio and distal colon cancer

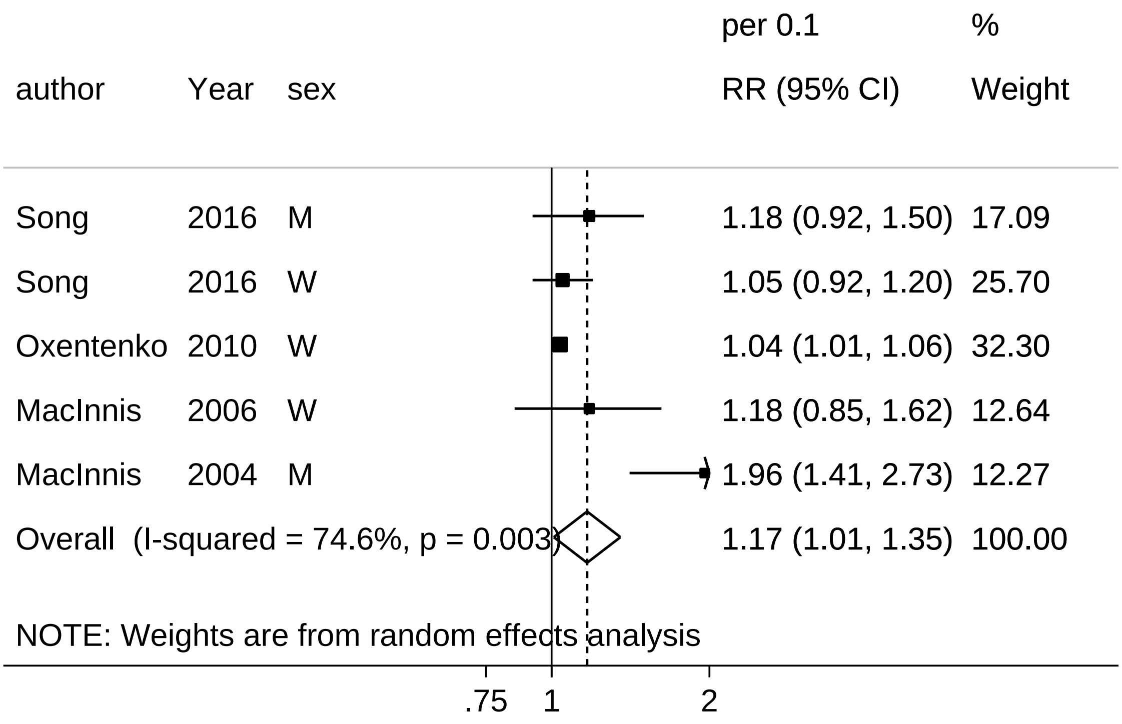

.**Figure20.**

A: waist to hip ratio and rectal cancer, per 0.1 unit

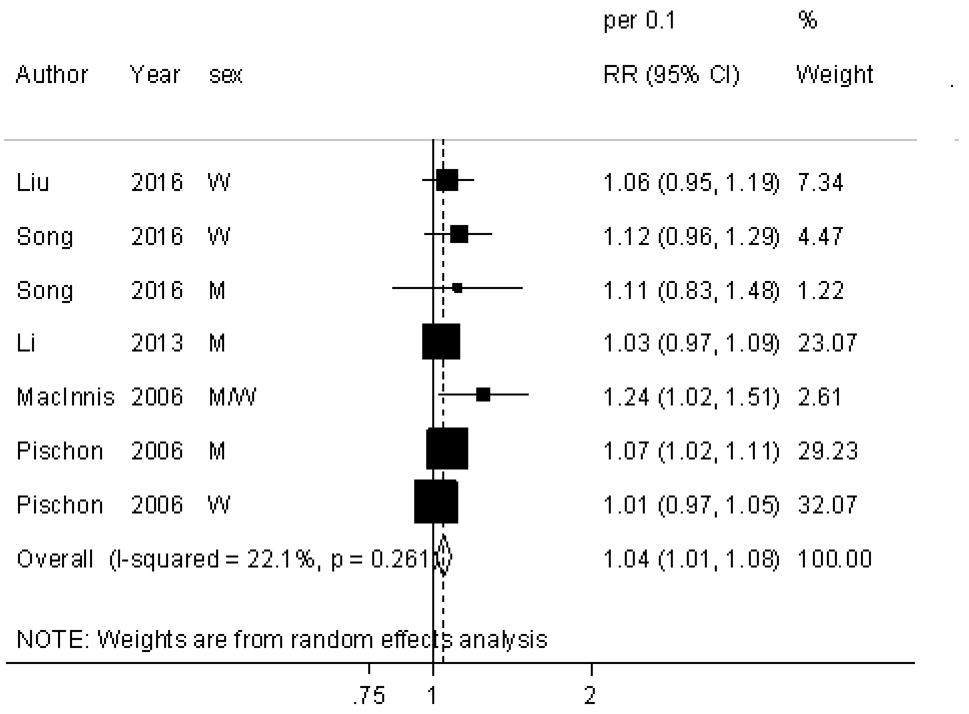

B: waist to hip ratio and rectal cancer, nonlinear dose-response

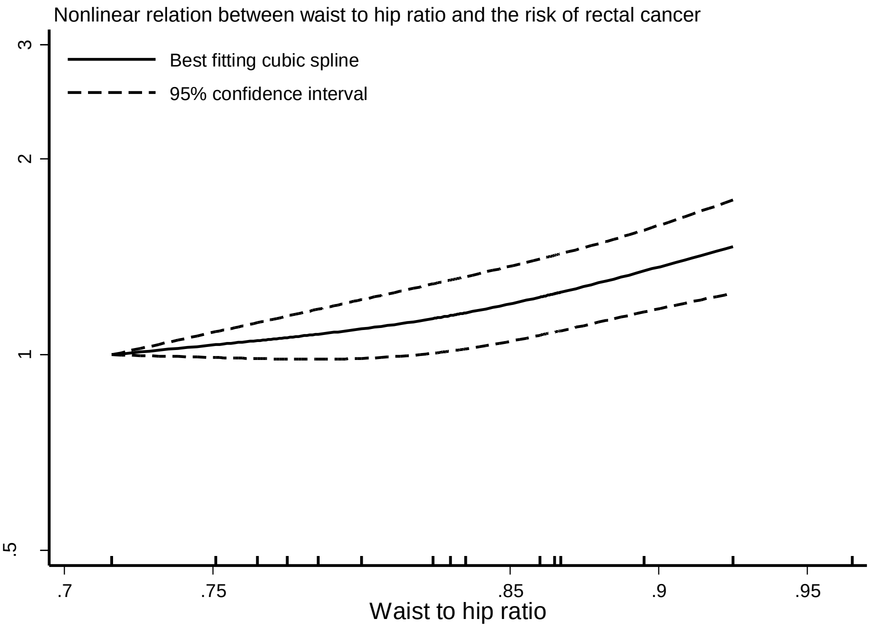

Supplement: Supplementary file 1 — Supplementary material 1 (PDF 939 KB) [file 394_2017_1557_MOESM1_ESM.pdf]
